# An Agonist/Antagonist Photo‐Switchable Vitamin D Mimetic Enables Bidirectional Optical Control of VDR

**DOI:** 10.1002/anie.9726457

**Published:** 2026-05-11

**Authors:** Xiu Ge, Sabine Willems, Francesco Melfi, Tufan Mukhopadhyay, Johannes Morstein, Jordan Artzy, Loris Knümann, Giorgia Sbriccoli, Jörg Pabel, Julian A. Marschner, Dirk Trauner, Daniel Merk

**Affiliations:** ^1^ Department of Pharmacy Ludwig‐Maximilians‐University (LMU) Munich Munich Germany; ^2^ Department of Pharmacy “G. d'Annunzio” University of Chieti‐Pescara Chieti Italy; ^3^ Department of Chemistry New York University New York New York USA; ^4^ Department of Chemistry University of Pennsylvania Philadelphia Pennsylvania USA

**Keywords:** azobenzene photoswitches, photopharmacology, transcription factor, vitamin D receptor

## Abstract

The secosteroid vitamin D (vitD) is essential for human health, maintaining bone metabolism, calcium homeostasis, and immune function via the vitamin D receptor (VDR). VDR activation has complex and cell‐specific effects in multiple tissues and spatial control of VDR activity is desirable. Here, we developed vitD mimetics bearing a photo‐switchable azobenzene motif to achieve spatiotemporal resolution in VDR activation. Structure‐guided tuning of regiochemistry and H‐bonding motifs provided a potent and selective vitD mimetic that can be reversibly switched in cellulo between agonist and antagonist states with light. In a model of osteogenesis, this unique bidirectional precision tool mediated light‐dependent osteogenic and osteolytic effects.

## Graphical abstract

Vitamin D exhibits complex and cell‐specific biological effects in multiple tissues via activation of the ligand‐sensing transcription factor vitamin D receptor (VDR). The photo‐switchable vitamin D mimetic **10** transitions between VDR antagonist (*E*) and agonist (*Z*) activity upon illumination and offers unprecedented bidirectional optical control over VDR activity which translated into light‐dependent osteogenic and osteolytic effects in vitro.

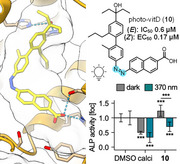

## 1 Introduction

**FIGURE 1:**
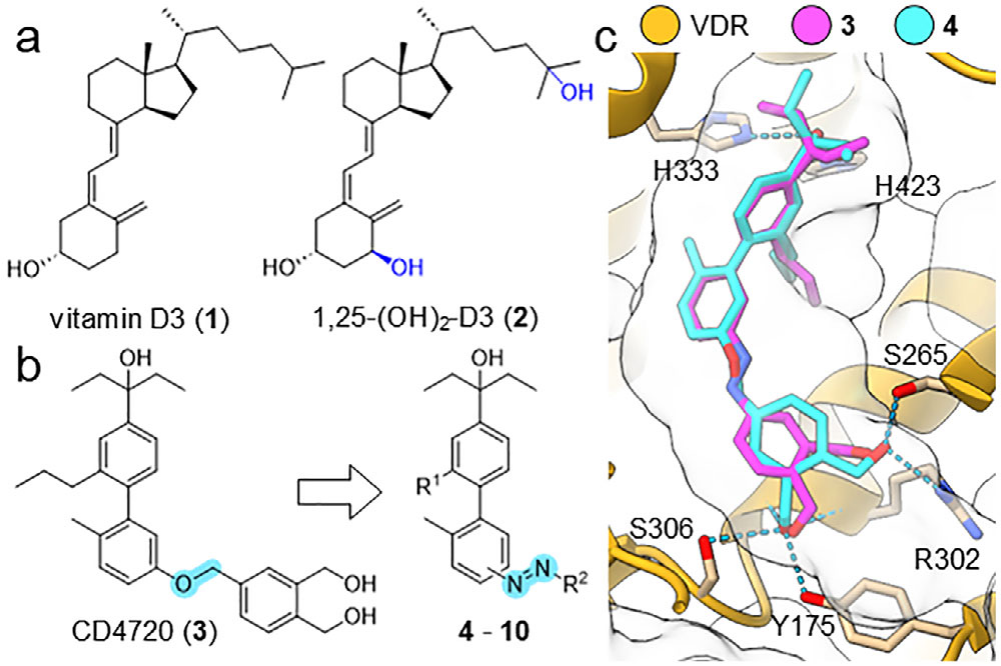
(a) Chemical structures of the natural vitamin D3 (**1**) and the hormonally active metabolite **2** formed by enzymatic hydroxylation in 1‐ and 25‐positions (blue). (b) Basic design of photo‐switchable VDR agonists (**4**‐**10**) by azologization [22] of the non‐secosteroidal VDR ligand **3**. (c) Binding mode of **3** (cyan) in the VDR ligand binding site (pdb ID 4g1d [21]). **3** displays optimal shape complementarity with the pocket and engages H‐bonds (blue dotted lines) with S265 and S306 via its bis(hydroxymethyl)benzene motif. The flexible benzyloxy linker is critical to enable proper orientation for H‐bonding. The predicted binding mode of (*E*)‐**4** (magenta) aligned well with the co‐crystallized ligand **3**.

Vitamin D (**1**, Figure 1a) is an essential molecule for human health with hormone‐like activity in the regulation of calcium homeostasis, bone metabolism, and immune function [1, 2, 3]. Its hormonally active form 1,25‐dihydroxyvitamin D (**2**, calcitriol), generated by double enzymatic hydroxylation, activates the vitamin D receptor (VDR), a ligand‐sensing transcription factor controlling gene expression in response to ligand‐binding via more than 10 000 loci in the human genome [2, 3, 4]. It regulates intestinal calcium absorption via induction of calcium channels and binding proteins that facilitate transcellular diffusion [2], and modulates osteoblast differentiation, extracellular matrix production, and mineralization in bone [5]. In immune cells, VDR is involved in the production of cytokines, the expression of co‐stimulatory molecules, and the regulation of immune cell phenotype and differentiation with an overall immune modulatory effect blocking the adaptive and promoting the innate immune response [2, 6, 7, 8]. VDR signaling is also linked to malignancies and low vitamin D levels are associated with a higher risk for, e.g., hematological, breast, colon, and prostate cancer [4]. Pharmacological VDR modulation may hence offer access to new therapies, e.g., in bone diseases, inflammatory disorders, and cancer [6, 7, 8, 9, 10, 11, 12], but the biological effects of vitamin D are complex, multifaceted, and cell‐specific. Activation of the natural vitamin to the hormonally active species underlies intense physiological regulation [2] and systemic VDR activation with synthetic VDR agonists lacking this natural control mechanism is associated with adverse effects [10].

Local VDR activation, e.g., in psoriatic lesions [13] or cancer, could mimic vitamin D effects at relevant sites while sparing the pleiotropic actions in other tissues. Such spatial control of drug bioactivity can be achieved with photo‐switchable molecules that exhibit different target affinities in different light‐induced configurations. This concept has been corroborated in photopharmacology for several macromolecular drug targets and enabled remarkable precision in the modulation of biological processes [14, 15, 16]. Nuclear receptors have also been addressed in photopharmacology with photohormones, e.g., targeting ER [17], FXR [18] and PPARs [19, 20]. Here, we established bidirectional optical control of the VDR which is unprecedented in photopharmacology. Structure‐guided design provided photo‐vitD (**10**) which exhibits potent VDR agonism (EC_50_ 0.17 µM) after activation with light ((*Z*)‐isomer) while the (*E*)‐state blocks VDR activity (IC_50_ 0.6 µM). In a cellular model of osteogenesis, the two photo‐vitD isomers exerted light‐dependent osteogenic or osteolytic effects corroborating it as high precision tool for bidirectional optical control of the VDR.

## 2 Results and Discussion

Our design approach to a light‐activated VDR agonist was based on the scaffold of CD4720 [21] (**3**; Figure 1b) which has been developed as a non‐secosteroidal VDR agonist. **3** binds to the L‐shaped hydrophobic VDR ligand binding pocket with optimal shape‐complementarity and engages key polar interactions with Ser265/Ser306 in the distal part of the molecule and with His333/His423 at the head hydroxy group (pdb ID: 4g1d; Figure 1c), which are also formed by the hormonally active vitamin D metabolite **2** [23]. The flexibility of the benzyloxy motif in **3** is critical to allow proper orientation of the bis(hydroxymethyl)benzene moiety for hydrogen bonding. This region, therefore, seemed ideally suited to incorporate an azobenzene photo‐switch. Modeling of the azologue **4** of **3** supported this design hypothesis (Figure 1c).

**SCHEME 1:**
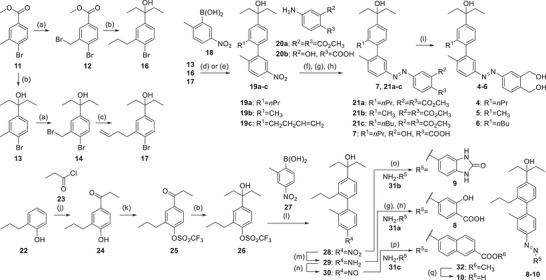
Synthesis of **4**–**10**. Reagents & Conditions: (a) NBS, (PhCOO)_2_, MeCN, 82°C, 16 h, 42%–79%; (b) EtMgBr, THF, 0°C‐r.t., 3–20 h, 28%–78%; (c) allylmagnesium bromide (**15**), THF, r.t., 16 h, 81%; (d) Pd(dppf)Cl_2_•CH_2_Cl_2_, K_3_PO_4_, 1,4‐dioxane/H_2_O, 82°C, 20 h, 36%–83%; (e) Pd(PPh_3_)_4_, Na_2_CO_3_, 1,4‐dioxane/MeOH, 90°C, 24 h, 55%; (f) Pd/C, H_2_, EtOAc/MeOH, r.t., overnight; (g) anilines **20a‐b, 31a**, Oxone, CH_2_Cl_2_/H_2_O, r.t., 3–18 h; (h) AcOH, r.t., 12 h‐3 d; (over three steps: f–h, 10%–70%); (i) LiAlH_4_, THF, 0°C‐r.t., 3–4 h, 25%–47%; (j) CF_3_SO_3_H, 0°C‐r.t., overnight, 45%; (k) (CF_3_SO_2_)_2_O, toluene, 0°C‐r.t., 3 h, 77%; (l) XPhosPd G2, K_2_CO_3_, toluene/EtOH/H_2_O, 85°C, 1 h, 92%; (m) Zn, NH_4_Cl, THF/MeOH/H_2_O, r.t.‐reflux, 6 h, 92%; (n) Oxone, CH_2_Cl_2_/H_2_O, r.t., 18 h, 48%; (o) **31b**, KOH, EtOH/H_2_O, μw irr., 120°C, 30 min, 6.3%; (p) **31c**, KOH, DMF, r.t., 10 min, 22%; (q) LiOH, THF/H_2_O, 45°C, 4 h, 44%.

Based on these observations, we prepared a series of CD4720 derivatives comprising a diazene linker and explored the bis(hydroxymethyl)benzene region and linker regiochemistry for a light‐activatable VDR agonist. The compounds were obtained via two routes according to Scheme 1. The first approach commenced with the preparation of 3‐alkyl‐4‐bromophenylpent‐3‐ol derivatives **13**, **16**, and **17** from methyl 4‐bromo‐3‐methylbenzoate (**11**), which was treated with ethylmagnesium bromide to install the pentanol motif in **13**. Radical bromination of **13** on the methyl substituent (**14**) and reaction with allylmagnesium bromide then afforded **17** bearing a but‐3‐ene side‐chain. The corresponding propyl analogue **16** was obtained via the same strategy but in inverted order with initial bromination of **11** to **12** and subsequent alkylation and installation of the pentanol group using ethylmagnesium bromide to obtain **16** in one step. The aryl bromides **13**, **16**, and **17** were then used for the Suzuki reaction with 2‐methyl‐5‐nitrophenylboronic acid (**18**) to yield **19a‐c**. Hydrogenation of the nitro group (and in case of **19c**, the butenyl motif) followed by Baeyer–Mills reaction with the anilines **20a‐b** afforded the azobenzene derivatives **7** and **21a‐c**. The bis(hydroxymethyl)phenyl derivatives **4**–**6** were obtained by reduction of the corresponding bis(methoxycarbonyl) analogues **21a‐c**.

For the synthesis of azobenzenes **8**–**10**, the 3‐propylphenylpentanol building block for the Suzuki reaction was prepared from 2‐propylphenol (**22**) which was acylated in a Friedel–Crafts reaction to **24**, and triflated to **25**. Reaction with ethylmagnesium bromide to install the pentanol motif in **26** and subsequent Suzuki reaction with 2‐methyl‐4‐nitrophenylboronic acid (**27**) afforded the building block **28** for azobenzene synthesis. The benzimidazolone derivative **9** was directly prepared from the nitrobenzene **28** by reaction with the aminobenzimidazolone **31b** under alkaline conditions. For the synthesis of **8**, **28** was reduced to the corresponding aniline and then reacted in a Baeyer–Mills reaction with 4‐amino‐salicylic acid (**31a**). To obtain **10**, the aniline building block **29** was oxidized to the nitroso analogue and then treated with methyl 6‐amino‐2‐naphthoate (**31c**) under alkaline conditions. The resulting azobenzene **32** was deprotected with LiOH to yield **10**.

**FIGURE 2:**
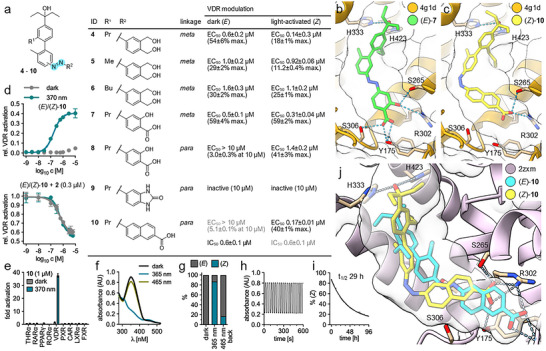
Development and characterization of the light‐activated vitamin D mimetic photo‐vitD (**10**). (a) Structure activity relationship of photo‐switchable VDR agonists **4**–**10**. Compounds were tested in a cellular Gal4‐VDR hybrid reporter gene assay. Relative activation is normalized to the effect of **2** (1 µM) set as 100% activation. The (*Z*)‐states of the compounds were maintained with a Cell‐Disco [24] (370 nm). Data are the mean±S.E.M., *n*≥3; inactive – no statistically significant activation at the indicated concentration. Data in agonist (EC_50_) and antagonist mode (IC_50_; vs. 0.3 µM **2**) are shown for (*E*)‐ and (*Z*)‐**10**. The corresponding dose‐response curves are shown in (d). (b) Predicted binding mode of (*E*)‐**7** (green) in the VDR ligand binding site (pdb ID 4g1d [21]). Docking suggested that replacement of the bis(hydroxymethyl)benzene motif (**4**) by a salicylic acid (**7**) could enable additional contacts with the polar pocket forming residues (Y175, S265, R302, S306). (c) Predicted binding mode of (*Z*)‐**10** (yellow) in the VDR ligand binding site. The altered regiochemistry conveys strong preference for the light‐induced (*Z*)‐configuration and the extended naphthoic acid motif enables a strong ionic contact to R302. (d) Dose‐response curves of (*E*)‐ (dark) and (*Z*)‐**10** (370 nm) in the Gal4‐VDR reporter gene assay in agonist (top) and antagonist mode (bottom, with 0.3 µM calcitriol (**2**)). (*Z*)‐**10** activated VDR; (*E*)‐**10** and (*Z*)‐**10** antagonized VDR activation by 0.3 µM calcitriol (**2**). (*Z*)‐**10** was maintained with a Cell‐Disco [24] (370 nm). Data are the mean±S.E.M. relative VDR act. vs. 0.3 µM calcitriol (**2**), *n*≥3. (e) Activity profile of **10** (1 µM) on nuclear receptors showing exclusive VDR activation by the light‐activated state. **10** was tested in a panel of uniform Gal4‐NR hybrid reporter gene assays. (*Z*)‐**10** was maintained with a Cell‐Disco [24] (370 nm). Data are the mean±SD, *n* = 3. (f) Representative UV/Vis spectra of **10** as (*E*)‐ (dark) and (*Z*)‐states (PSS 365 nm), and after switching back to (*E*) with 465 nm. (g) (*E*)/(*Z*)‐ratios of **10** in the dark‐adapted state, after illumination at 365 nm for 5 min and after switching back from mostly‐(*Z*) with 465 nm for 5 min. (h) **10** could be rapidly and reversibly switched between the (*E*)‐ and (*Z*)‐states over multiple cycles by alternating irradiation at 365 and 465 nm. Absorbance was measured at 351 nm with a 25 µM solution in DMSO, one cycle is 30 sec. (i) Thermal relaxation of (*Z*)‐**10** with a half‐life of 29 h at room temperature (∼21°C). **10** was pre‐illuminated at 365 nm for 30 min and then kept in the dark. (*E*)/(*Z*) ratios were measured by NMR. (j) Binding mode prediction of (*E*)‐ and (*Z*)‐**10** to the VDR‐antagonist complex 2zxm [25] (residues numbered according to [21]). (*Z*)‐**10** (yellow) forms an agonist‐like binding mode with several polar interactions at both ends of the pocket. (*E*)‐**10** (cyan) is also anchored at the distal end of the pocket by an H‐bond network with R302 and the D176 backbone but due to the (*E*)‐configuration, the β‐naphthoic acid residue protrudes deeper and the pentan‐3‐ol motif rotates away from H333/H423. Therefore, (*E*)‐**10** lacks stabilizing/activating contacts with the H333/H423 region [21, 25, 26, 27] rationalizing its antagonist properties.

In line with the modeling results (Figure 1c), the azobenzene analogue **4** of **3** retained strong VDR agonism with sub‐micromolar potency (Figure 2a) indicating that the photo‐switchable motif was tolerated and did not disrupt binding. Additionally, we detected a slight, 4‐fold activity difference between the dark‐adapted (*E*)‐ and the light‐induced (*Z*)‐isomers of **4** which further supported the scaffold's suitability to develop a photo‐switchable vitamin D mimetic, but structural modifications were needed to establish sufficient preference for one configuration. Shortening of the 2’‐alkyl chain to methyl (**5**) and its extension to butyl (**6**) were not favored both in terms of potency and preference, and we hence kept the previously optimized [21] biphenyl residue and its substitution pattern fixed.

Modeling indicated that additional (ionic) interactions could be accessible via incorporation of an acidic motif in the bis(hydroxymethyl)benzene region of **4** (Figure 2b). The salicylic acid analogue **7** indeed gained in activation efficacy of the (*Z*)‐configuration and retained sub‐micromolar potency but the activity difference between the (*E*)‐ and (*Z*)‐configurations remained low. Further binding mode inspection suggested that a different regiochemistry with the diazene in para‐position of the biphenyl residue might convey a stronger preference for the (*Z*)‐isomer (Figure S1a). The para‐analogue **8** of **7** indeed displayed pronounced VDR agonism of the light‐induced (*Z*)‐configuration while the dark‐adapted (*E*)‐isomer was markedly less active. **8** thus exhibited the desired light‐dependent VDR agonism but displayed only intermediate potency.

We reasoned that the altered regiochemistry of **8** might result in an inappropriate placement of the polar salicylic acid head for hydrogen bonding with Ser265/Ser306 and hence explored other motifs on the diazene. A benzimidazolone (**9**) appeared promising according to docking (Figure S1b) but failed to activate VDR in both the (*E*)‐ and (*Z*)‐configurations, while extension of the salicylic acid (**8**) to the naphthalene carboxylate **10** (Figure 2c) eventually provided the desired light‐induced VDR agonism with strong potency. **10** activated VDR with an EC_50_ value of 0.17 µM in the (*Z*)‐configuration, while the (*E*)‐isomer failed to activate VDR (Figure 2d) but unprecedentedly competed with calcitriol (**2**) and acted as VDR antagonist blocking the calcitriol‐induced VDR activation with an IC_50_ value of 0.6 µM.

VDR agonists typically form networks of hydrogen bonds with polar residues (Tyr175/Ser265/Arg302/Ser306 and His333/His423; numbering according to [21]) at both ends of the ligand binding site as illustrated by **3** (Figure 1c) [21, 26]. Contacts to His333/His423 located in helices 6 and 11 are thought to be critical for activation as these residues communicate with helix 12 via an allosteric network to stabilize the activation function (AF2) in an active conformation. Antagonist VDR ligands deviate from this pattern by reduced polar contacts to His333/His423, thus mediating less stabilization in this region of the VDR ligand binding domain but blocking the pocket [25, 26, 27]. The predicted binding modes of (*E*)‐ and (*Z*)‐**10** to VDR (Figure 2j) aligned well with this mechanistic model: while the agonist‐like binding of (*Z*)‐**10** involving polar contacts with both ends of the ligand binding site was reproduced even by docking into the VDR‐antagonist complex 2zxm [25], (*E*)‐**10** displayed a significantly shifted and rotated binding mode lacking interactions with His333/His423. Together, the in vitro activity profile and modeling results thus suggest that (*E*)‐**10** binds to VDR without stabilizing AF2 but competitively blocking agonist binding.

In vitro characterization of **10** revealed strong selectivity with no activity on nuclear receptors related to VDR in both (*E*)‐ and (*Z*)‐configuration (Figure 2e). The dark‐adapted (*E*)‐**10** displayed efficient photo‐switching at 365 nm to a photostationary state (PSS) of 85:15 (*Z*):(*E*) for activation to a VDR agonist and illumination at 465 nm enabled light‐induced inactivation back to the antagonist (*E*)‐**10** (Figure 2f,g). **10** could be repeatedly and rapidly switched between (*E*)‐ and (*Z*)‐states with no fatigue (Figure 2h) and the light‐induced (*Z*)‐**10** displayed a thermal relaxation half‐life of 29 h (Figure 2i). These favorable photophysical properties as well as the strong, opposing VDR modulator potency in the (*E*)‐ and (*Z*)‐configurations thus rendered **10** as optically controllable vitamin D mimetic suitable as high‐precision tool.

**FIGURE 3:**
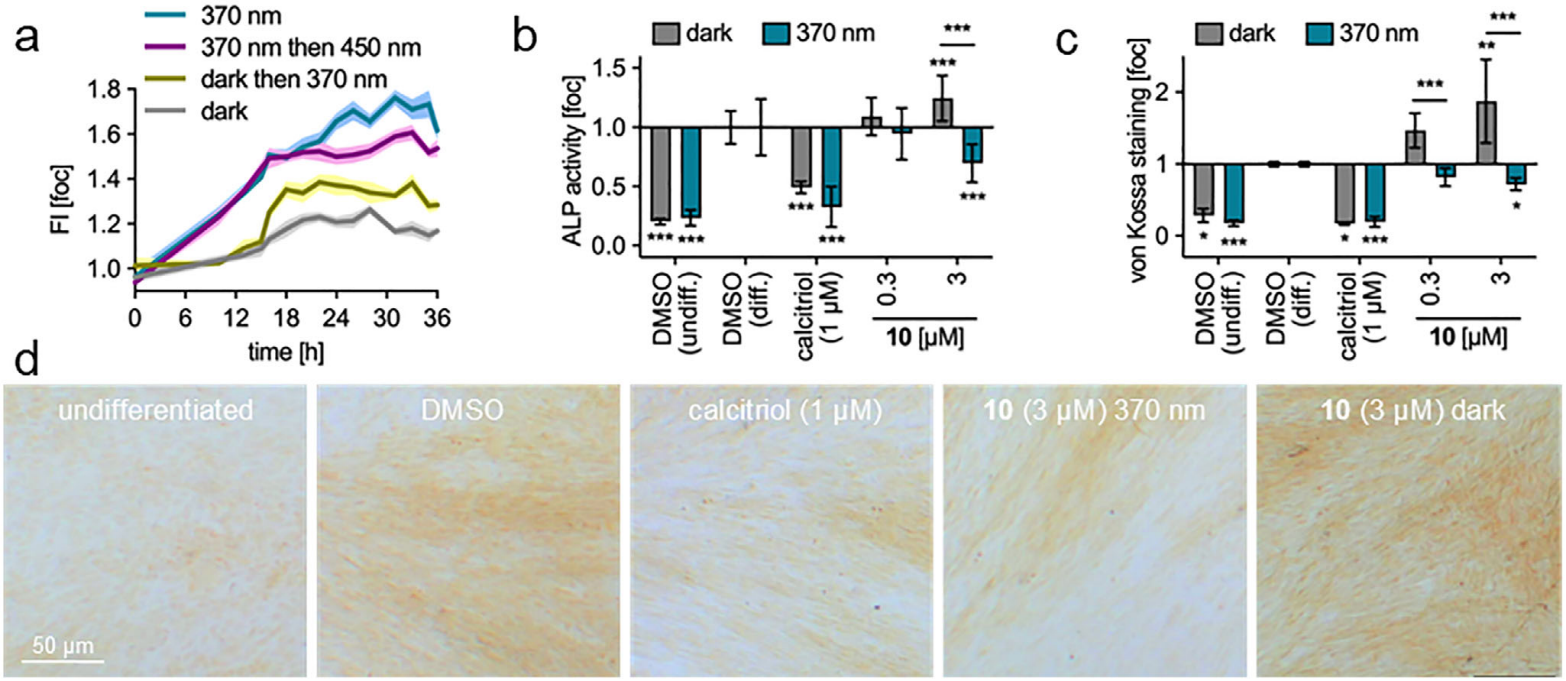
Optical control of VDR activity with photo‐vitD (**10**). (a) **10** (1 µM) enabled light‐dependent in‐cell activation of VDR in a fluorescent (mCherry) reporter gene assay. Gray curve: dark; blue curve: Cell‐Disco (370 nm; 100 ms/10 s); yellow curve: dark for 10 h, then Cell‐Disco (370 nm); magenta curve: Cell‐Disco (370 nm) for 10 h, then Cell‐Disco (450 nm). Lines are the mean, shadows are S.E.M.; *n* = 6. (b–d) Phenotypic model of bone formation using human fibroblasts (ASC52telo hTERT cells) and cyclic treatment with dexamethasone, 2‐phospho‐L‐ascorbic acid, and glycerol 2‐phosphate over 15 days to induce osteogenesis. Alkaline phosphatase (ALP) activity (b) and von Kossa staining of hydroxyapatite (c; representative photomicrographs in d) were used as markers of osteogenesis. Experiments were performed in the dark (gray bars) and using a Cell‐Disco (370 nm, 100 ms/10 s; blue bars). Data are the mean±SD, *n* = 6. * *p*<0.05, ** *p*<0.01, *** *p*<0.001 (vs. respective dark or illuminated DMSO (diff.) ctrl or as indicated; ANOVA with Dunnett's multiple comparisons test).

To evaluate the potential of **10** for in‐cell control of VDR activity, we determined its light‐dependent effect over time (Figure 3a) using VDR‐induced expression of the fluorescent protein mCherry [20] as reporter for non‐invasive monitoring. Under illumination with a Cell‐Disco [24] (370 nm, 100 ms/10 s) to maintain **10** in VDR activating (*Z*)‐state, mCherry fluorescence constantly increased (Figure 3a, blue curve) indicative of enhanced VDR activity. When **10** was switched back to the (*E*)‐state with 450 nm light (Cell‐Disco), the increase in mCherry fluorescence ceased (Figure 3a, magenta curve). Without illumination, **10** caused no increase in fluorescence (Figure 3a, gray curve) but could be switched on with 370 nm light to induce delayed mCherry expression (Figure 3a, yellow curve). These time courses of VDR‐induced mCherry fluorescence in cellulo demonstrated that the activity of **10** as VDR modulator could be switched with light in living cells corroborating optical control of VDR.

Photo‐vitD (**10**) thus appeared suitable for application as tool for VDR control in complex phenotypic settings. In a cellular model of osteogenesis from human fibroblasts (ASC52telo hTERT cells), **10** revealed a profound, light‐dependent impact on bone formation markers (Figure 3b–d). Treatment of ASC52telo hTERT cells with dexamethasone, 2‐phospho‐L‐ascorbic acid, and glycerol 2‐phosphate in six cycles over 15 days resulted in osteogenesis as evident from markedly enhanced alkaline phosphatase (ALP) activity (Figure 3b) and von Kossa staining positive area (Figure 3c,d) compared to undifferentiated cells. The natural VDR agonist calcitriol (**2**) counteracted bone formation in line with previous reports [28, 29] of pro‐resorptive activity. Light‐activated photo‐vitD (i.e., (*Z*)‐**10**) acting as VDR agonist resembled the effects of calcitriol with weaker efficacy (matching its partial agonist profile), while (*E*)‐**10** acting as VDR antagonist promoted osteogenesis as evident from increased ALP activity and von Kossa positive area. Photo‐vitD (**10**) thus enabled bidirectional optical control of osteogenesis in cellulo based on its ability to switch between agonist and antagonist states.

## 3 Conclusion

The rationally designed, light‐controlled vitamin D mimetic photo‐vitD (**10**) enables unprecedented optical control of the vitamin D receptor, a potent regulator of human physiology with complex and multifaceted roles in many cell types [2, 4]. Its bidirectional activity profile with light‐dependent transition between antagonism and agonism is unique among molecular photo‐switches and offers significant advantages in biological applications. As a high‐precision chemical tool, **10** may help dissect VDR mediated effects through selective and spatially resolved receptor activation and inhibition, which cannot be achieved with traditional VDR ligands. Additionally, spatial control of VDR activity may be therapeutically attractive: as VDR activation can have strong impact on cell fate, the metabolic activation of vitamin D to the hormonally active species underlies intense physiological control [2, 3]. Loss of this physiological regulation can be detrimental as evident from adverse effects of synthetic VDR agonists [10] which limit their application despite therapeutic promise of VDR activation in severe conditions such as cancer, autoimmune disorders, and inflammatory skin diseases [30]. Local VDR activation with a photo‐switchable agonist at easily accessible sites of action like psoriatic lesions in the skin could hence be a very promising field for applied photopharmacology [13].

## Author Contributions

**Xiu Ge**: investigation, formal analysis, writing – review and editing. **Sabine Willems**: investigation, formal analysis, conceptualization, data curation, writing – review and editing, visualization, validation. **Francesco Melfi**: investigation. **Tufan Mukhopadhyay**: investigation. **Johannes Morstein**: investigation. **Jordan Artzy**: investigation. **Loris Knümann**: investigation. **Giorgia Sbriccoli**: investigation. **Jörg Pabel**: formal analysis, validation, writing – review and editing. **Julian A. Marschner**: investigation, formal analysis, data curation, validation, methodology, writing – review and editing. **Dirk Trauner**: conceptualization, supervision, methodology, writing – review and editing. **Daniel Merk**: conceptualization, writing – original draft, supervision, data curation, project administration, methodology, validation, visualization, writing – review and editing.

## Conflicts of Interest

The authors declare no conflicts of interest.

## Supporting information




**Supporting File**: Figure S1, synthetic procedures and analytical characterization of **4**–**10**, methods for photophysical profiling and in vitro assays, and computational procedures. The authors have cited additional references within the Supporting Information [31, 32, 33, 34, 35, 36, 37, 38, 39].

## Data Availability

The data that supports the findings of this study are available in the supplementary material of this article.
