# Supplementary material for: An Agonist/Antagonist Photo‐Switchable Vitamin D Mimetic Enables Bidirectional Optical Control of VDR
Source: Angew Chem Int Ed Engl. 2026 May 11;65(29):e9726457. doi: 10.1002/anie.9726457 (PMC13360702; doi:10.1002/anie.9726457)
Supplement: Supplementary file 1 — Supporting File: Figure S1, synthetic procedures and analytical characterization of 4–10, methods for photophysical profiling and in vitro assays, and computational procedures. The authors have cited additional references within the Supporting Information [31, 32, 33, 34, 35, 36, 37, 38, 39]. [file ANIE-65-e9726457-s001.pdf]

- Supporting Information -

An Agonist/Antagonist Photo-Switchable Vitamin D Mimetic Enables Bidirectional Optical Control of VDR

Xiu Ge,[a]#, Sabine Willems,[a]#, Francesco Melfi,[a,b], Tufan Mukhopadhyay,[c] Johannes Morstein,[c] Jordan Artzy,[d] Loris Knümann,[a] Giorgia Sbriccoli,[a] Jörg Pabel,[a] Julian A. Marschner,[a] Dirk Trauner[c,d]*, Daniel Merk[a]*

[a] X. Ge, Dr. S. Willems, F. Melfi, L. Knümann, G. Sbricolli, Dr. J. Pabel, Dr. J.A. Marschner, Prof. Dr. D. Merk
Department of Pharmacy
Ludwig-Maximilians-University (LMU) Munich
Munich, Germany
E-mail: daniel.merk@cup.lmu.de

[b] F. Melfi
Department of Pharmacy
"G. d'Annunzio" University of Chieti-Pescara
Chieti, Italy

[c] Dr. T. Mukhopadhyay, Dr. J. Morstein, Prof. Dr. D. Trauner
Department of Chemistry
New York University
New York, NY, USA

[d] J. Artzy, Prof. Dr. D. Trauner
Department of Chemistry
University of Pennsylvania
Philadelphia, PA, USA
E-mail: dtrauner@upenn.edu

# X. Ge and S. Willems contributed equally to this study

Table of Contents

Supplementary Figures 2
Chemistry 3
Photophysical characterization 13
In vitro assays 14
Computational methods 17
NMR spectra of **4-10** 18
Supplementary references 25

Supplementary Figures

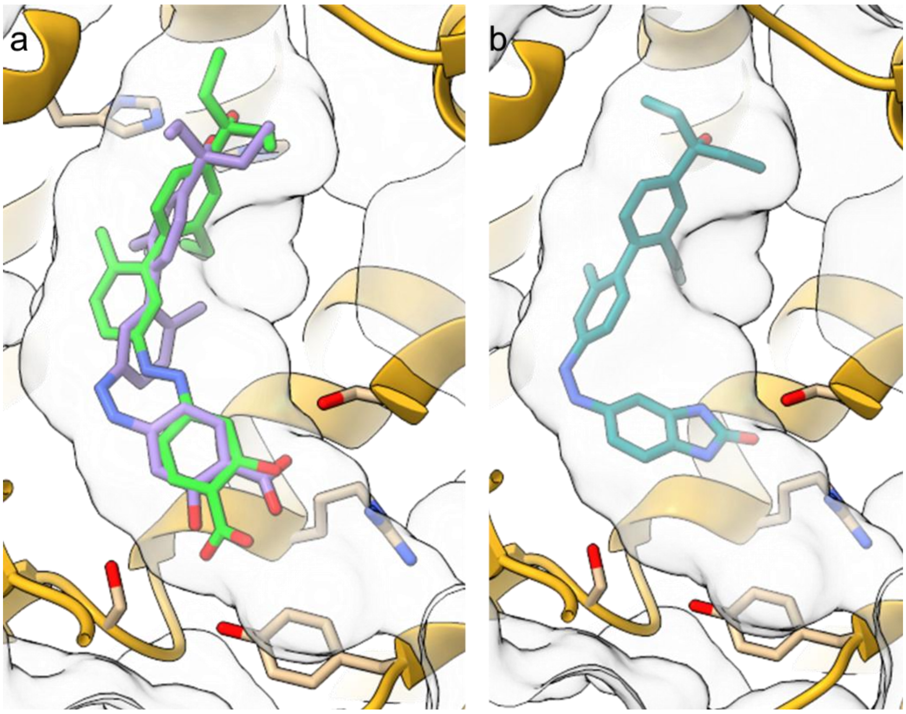

Figure S1. Predicted binding modes of **8** (violet, a) and **9** (teal, b) in the VDR ligand binding site (pdb ID: 4g1d[1]). (a) Docking suggested that **8** bearing the diazene linker in para-position of the biphenyl motif could convey (*Z*)-preference. The meta-analogue **7** (green) is shown for comparison. (b) Docking supported potential of the benzimidazolone motif in **9** for favorable VDR binding.

Chemistry

General. All chemicals were of reagent grade, purchased from commercial sources (e. g., Sigma-Aldrich, TCI, BLDpharm) and used without further purification unless otherwise specified. All reactions were conducted in common glassware under air unless otherwise specified and in absolute solvents purchased from Merck-Sigma-Aldrich or Thermo scientific. Other solvents, especially for work-up procedures, were of reagent grade or purified by distillation (cyclohexane, ethyl acetate (EtOAc), ethanol (EtOH). Reactions were monitored by thin layer chromatography (TLC) using fluorescent dye-coated silica gel 60 on aluminum sheets by Merck and visualized under ultraviolet light (254 nm). Purification was performed by automated flash column chromatography (aFCC) on an Interchim puriFlash® XS520Plus system (Advion, Ithaca, NY, USA) on pre-packed silica cartridges from BUCHI FlashPure EcoFlex (PF-15SIHPF0025, PF-50SIHP-F0012, PF-50SIHP-F0025 or PF-50SIHP-F0040) dry loading the crude product on silica gel and a gradient of cyclohexane to ethyl acetate. Reversed-phase automated flash column chromatography (RP-CC) was performed on a puriFlash® XS520Plus (Advion) using C18HP columns (PF-15C18HP-F0012 or PF-15C18HP-F0025) from Interchim and a gradient of H_2O with 25 % acetonitrile (MeCN) to 100 % MeCN (HPLC gradient grade) and by dry loading the crude product on celite. Microwave-assisted synthesis was performed using a Biotage® Initiator™ (2011) equipped with temperature and pressure control in a 10 mL sealed vial (temperature: 60-250 °C (140-482 °F); temperature increase 2-5 °C/s (36-41 ˚F/s); pressure range 0-20 bar (2 MPa, 290 PSI); power range 0-300 W at 2.45 GHz). The internal temperature of the vial was monitored by an infrared sensor. ^{1}H and ^{13}C NMR spectra were recorded at room temperature (r.t.) on Bruker AVANCE III HD 400, Bruker AVANCE III HD 500 or Bruker AVANCE NEO 600 MHz spectrometers (Bruker Corporation, Billerica, MA, USA). Chemical shifts (δ) are reported in parts per million (ppm). The NMR spectra were calibrated using the proton or carbon signals of residual nondeuterated solvent peaks (^{1}H-NMR: acetone-d_6: δ = 2.05 ppm; $CDCl_3$: δ = 7.26 ppm; DMSO-d_6: δ = 2.50 ppm; MeOD-d_4: δ=3.31 ppm, ^{13}C-NMR: acetone-d_6: δ = 206.26, 29.84 ppm; $CDCl_3$: δ = 77.16 ppm; DMSO-d_6: δ = 39.52 ppm; MeOD-d_4: δ = 49.0 ppm). The experimental uncertainty of the coupling constants is estimated to be 0.1 Hz; deviations of *J* values within this range are not considered significant. Signal multiplicity is reported as follows: s, singlet; brs, broad singlet; d, doublet; t, triplet; q, quartet; h, heptet; m, multiplet, dd, doublet of doublets; dt, doublet of triplets; td, triplet of doublets. Due to the trans/cis isomerization of some compounds containing an azobenzene functionality, more signals were observed in the ^{1}H and ^{13}C spectra than would be expected for the pure trans-isomer. Only signals for the major trans-isomer are reported. Quantitative ^{1}H NMR (qHNMR)[2] experiments were acquired at r.t. with 64 scans in a spectral range from −7.5 to 22.5 ppm relative to TMS using maleic acid (Lot#BCCK2148) or ethyl 4-(dimethylamino)benzoate (Lot#BCCL6166) as the internal standard. Pulse delay was set to 60 s, the acquisition time was 4 s (digital resolution = 0.05 Hz/point), and 2 dummy scans were performed before the acquisition. Alternatively, purity was tested by HPLC-UV-MS, using a Shimadzu (Kyoto, Japan) HPLC system (CNM-40 controller, LC-40D quaternary pump, SIL-

20A-HT autosampler, SPD-M40 photodiode array detector, LCMS-2020 mass spectrometer with ESI ion source) with a Zorbax SBAg column (3 x 100 mm, 3.5 μm, Agilent, protected with a 0.5 μm and a 0.2 μm frit) in combination with H_2O (+0.1%FA) (A) and MeCN (+0.1%FA) (B) as mobile phase at a flow rate of 0.5 mL/min. Low resolution MS spectra were recorded with an Advion expression™ CMS (Advion, Inc., Ithaca, NY, USA) using atmospheric pressure chemical ionization (APCI). High resolution MS analyses were performed on a Finnigan MAT 95 spectrometer (Thermos Fisher Scientific) using electrospray ionization (ESI). All final compounds for biological evaluation had a purity of >95% according to qHNMR or HPLC-MS.

General procedure A for reduction of esters. A flame-dried septa-fitted vial charged with a stirred solution of the respective ester **21a-c** (1.0 equiv.) in anhydrous THF (0.012-0.078 M) was cooled to 0 °C. A solution of 1 M (or 2 M) $LiAlH_4$ in THF (4-10 equiv.) was added dropwise at 0 °C. The mixture was stirred for 1-3.5 h at r.t. and the reaction was quenched with excess MeOH. The mixture was diluted with $CHCl_3$ or CH_2Cl_2, the combined organic layers were washed with 1 M NaOH (1×) and brine (1×), dried over Na_2SO_4 and filtered, and the solvent was evaporated under reduced pressure. The resulting oil was purified via column chromatography (SiO_2, gradient: 0-5% MeOH:CH_2Cl_2) to give **4-6**.

General procedure B for Grignard addition reaction. The respective ester **11-12** or ketone **25** (1.0 equiv.) and anhydrous THF (0.5-0.87 M) were added into a flame-dried Schlenk under Ar. The solution was cooled to 0 °C in an ice-water bath and the solution of ethyl magnesium bromide (EtMgBr, 1.0 M in THF, 1.3-3.5 equiv.) was added dropwise. The reaction was quenched at 0 °C with H_2O, the resulting mixture was extracted with EtOAc or Et_2O (3×) and the combined organic layers were washed with H_2O (2×) and brine (1×), dried over Na_2SO_4, filtered and concentrated under reduced pressure. The resulting residue was purified by column chromatography (SiO_2; gradient: 0-30% EtOAc in hexanes) to give **13** and **16** or by aFCC using 3%-10 % EtOAc in cyclohexane as gradient to obtain **26**.

General procedure C for Suzuki coupling. To a flame-dried vial was added the respective bromide (1.0 equiv.), (2-methyl-5-nitrophenyl)boronic acid (**18**, 1.1 equiv.), Pd(dppf)Cl_2·CH_2Cl_2 (0.043 equiv.), and K_3PO_4 (3.3 equiv.). The vial was capped and purged with Ar gas before addition of 1,4-dioxane (0.23 M), followed by H_2O (5.5 equiv.). The reaction mixture was heated to 80 °C and stirred overnight. The reaction mixture was cooled to r.t., diluted with EtOAc, and washed with H_2O (1×) and brine (1×). The organic layer was dried over Na_2SO_4, filtered, and concentrated under reduced pressure. The resulting crude oil was purified using column chromatography (SiO_2; gradient: 0-30% EtOAc in hexanes) to give **19a-b**.

General procedure D for palladium-carbon catalytic reduction reaction. The respective nitro-intermediate **19a-c** (1.0 equiv.) was dissolved in 2:1 EtOAc:MeOH (v:v, 10.5 mL total, 0.14-0.02 M) and the solution was degassed by purging with Ar or N_2 gas. Pd/C (10 wt%, 0.17 equiv.) was added, and the

suspension was briefly purged with hydrogen gas and left under an H_2 atmosphere (H_2 balloon) while stirred at r.t. overnight. The mixture was filtered through a pad of celite and washed with EtOAc, the filtrate was concentrated under reduced pressure. The crude product (the respective amine) was used for the Baeyer-Mills coupling without further purification.

General procedure E for Baeyer-Mills reaction. The respective aniline **20a-b**, **31a** (1.0–2.0 eq) was dissolved in CH_2Cl_2 (0.05-0.09M), the solution was treated with a solution of Oxone® ($2KHSO_5 \cdot KHSO_4 \cdot K_2SO_4$, 4.0-6.0 equiv.) in H_2O (0.21-0.44 M). The resulting biphasic mixture was stirred rapidly at r.t. for 3 h-overnight. The two layers were separated, and the organic phase layer was washed with 1 M HCl, saturated $NaHCO_3$ solution, and H_2O. The organic phase was dried over Na_2SO_4, filtered and concentrated to 1-5 mL under reduced pressure. A solution of the second aniline the reduced compounds **19a-c** using general procedure D and **29** (1.0 equiv.) in CH_2Cl_2 was added, followed by AcOH (0.06-0.1 M). CH_2Cl_2 was removed under reduced pressure, and the resulting mixture was purged briefly with N_2 or Ar and stirred at r.t. for 12 h-3 d with protection from light. AcOH was removed under reduced pressure, and, if necessary, the residue was azeotroped with toluene (2×2 mL). The residue was purified by flash column chromatography (SiO_2, gradient: 0-50% EtOAc in hexanes) to give **21a-c**, by flash column chromatography (SiO_2, 10% MeOH in CH_2Cl_2) to give **7** or by RP-CC with 40-100% MeCN in H_2O as gradient to give **8**.

*<u>Synthesis and analytical data of **4**-**10** and precursors</u>*

(*E*)-(4-{[4'-(3-Hydroxypentan-3-yl)-6-methyl-2'-propyl[1,1'-biphenyl]-3-yl]diazenyl}-1,2-phenylene)dimethanol (4). Synthesis was performed according to general procedure A using a solution of **21a** (25 mg, 0.048 mmol, 1.0 equiv.) in anhydrous THF (0.62 mL, 0.078 M) and a solution of $LiAlH_4$ in THF (1 M, 0.19 mL, 0.19 mmol, 4.0 equiv.) to give compound **4** as an orange oil (11 mg, 47%). ^1H NMR (400 MHz, $CDCl_3$): δ 7.87-7.85 (m, 1H), 7.85-7.81 (m, 2H), 7.74 (d, *J* = 2.1 Hz, 1H), 7.49 (d, *J* = 7.9 Hz, 1H), 7.40 (d, *J* = 8.1 Hz, 1H), 7.31 (d, *J* = 1.9 Hz, 1H), 7.22 (dd, *J* = 7.9, 2.0 Hz, 1H), 7.09 (d, *J* = 7.9 Hz, 1H), 4.82 (d, *J* = 2.2 Hz, 2H), 4.80 (d, *J* = 2.1 Hz, 2H), 2.51-2.30 (m, 2H), 2.12 (s, 3H), 1.97-1.80 (m, 4H), 1.45 (h, *J* = 7.5 Hz, 2H), 0.82 (t, *J* = 7.4 Hz, 6H), 0.77 (t, *J* = 7.3 Hz, 3H) ppm. ^{13}C NMR (101 MHz, $CDCl_3$): δ 152.7, 150.6, 145.0, 142.5, 142.0, 140.4, 140.3, 139.8, 138.4, 130.7, 130.5, 129.3, 126.4, 124.4, 123.5, 123.2, 122.9, 121.8, 63.9, 53.6, 35.5, 35.1, 35.0, 24.1, 20.3, 14.1, 8.1 ppm. HRMS (ESI): *m/z* calculated 461.2799 for $C_{29}H_{36}N_2O_3+H^+$, found 461.2800 $[M+H]^+$.

(*E*)-(4-{[4'-(3-Hydroxypentan-3-yl)-2',6-dimethyl[1,1'-biphenyl]-3-yl]diazenyl}-1,2-phenylene)dimethanol (5). Synthesis was performed according to general procedure A using a solution of **21b** (30 mg, 0.061 mmol, 1.0 equiv.) in anhydrous THF (0.79 mL, 0.078 M) and a solution of $LiAlH_4$ in

THF (1 M, 0.25 mL, 0.25 mmol, 4.0 equiv.) to give compound **5** as an orange solid (6.6 mg, 25%). ^{1}H NMR (400 MHz, $CDCl_3$): δ 7.87 (d, *J* = 2.0 Hz, 1H), 7.85-7.81 (m, 2H), 7.72 (d, *J* = 2.1 Hz, 1H), 7.50 (d, *J* = 8.0 Hz, 1H), 7.41 (d, *J* = 8.2 Hz, 1H), 7.30 (d, *J* = 1.9 Hz, 1H), 7.22 (dd, *J* = 7.9, 2.0 Hz, 1H), 7.11 (d, *J* = 7.9 Hz, 1H), 4.83 (s, 2H), 4.80 (s, 2H), 2.13 (s, 3H), 2.11 (s, 3H), 1.95-1.80 (m, 4H), 0.83 (t, *J* = 7.4 Hz, 6H) ppm. ^{13}C NMR (101 MHz, $CDCl_3$): δ 152.7, 150.8, 145.1, 142.6, 142.0, 140.3, 140.3, 138.8, 135.4, 130.7, 130.5, 129.0, 127.2, 123.8, 123.5, 123.2, 123.0, 122.2, 63.9, 35.1, 35.0, 20.3, 20.1, 8.1 ppm. HRMS (ESI): *m/z* calculated 433.2486 for $C_{27}H_{32}N_2O_3$+H^+, found 433.2485 $[M+H]^+$.

(*E*)-(4-{[2'-Butyl-4'-(3-hydroxypentan-3-yl)-6-methyl[1,1'-biphenyl]-3-yl]diazenyl}-1,2-phenylene)dimethanol (6). Synthesis was performed according to general procedure A using a solution of **21c** (12.5 mg, 0.0236 mmol, 1.0 equiv.) in anhydrous THF (2.0 mL, 0.12 M) and a solution of $LiAlH_4$ in THF (2 M, 118 µL, 0.236 mmol, 10.0 equiv.) to give compound **6** as a yellow-orange oil (4 mg, 36%). ^{1}H NMR (500 MHz, acetone-*d*$_6$): δ 8.02-8.01 (m, 1H), 7.86 (dd, *J* = 8.1, 2.2 Hz, 1H), 7.84-7.80 (m, 1H), 7.70-7.69 (m, 1H), 7.63 (d, *J* = 8.1 Hz, 1H), 7.51 (d, *J* = 8.2 Hz, 1H), 7.47-7.43 (m, 1H), 7.34 (dd, *J* = 7.9, 1.9 Hz, 1H), 7.13-7.08 (m, 1H), 4.82-4.77 (m, 4H), 3.60 (s, 1H), 2.57-2.35 (m, 2H), 2.14 (s, 4H), 1.91-1.80 (m, 4H), 1.43 (p, *J* = 7.6 Hz, 2H), 1.22-1.12 (m, 2H), 0.81-0.78 (m, 6H), 0.75-0.72 (m, 3H) ppm. ^{13}C NMR (151 MHz, acetone-*d*$_6$): δ 152.7, 151.5, 146.9, 143.9, 143.4, 141.8, 140.7, 140.1, 138.5, 131.6, 129.5, 129.0, 127.6, 124.3, 124.1, 122.7, 122.5, 122.1, 77.1, 62.4, 62.3, 36.1, 34.0, 33.6, 23.0, 20.2, 14.1, 8.4 ppm. HRMS (ESI): *m/z* calculated 475.2955 for $C_{30}H_{38}O_3N_2$+H^+, found 475.2945 $[M+H]^+$.

(*E*)-2-Hydroxy-4-{[4'-(3-hydroxypentan-3-yl)-6-methyl-2'-propyl[1,1'-biphenyl]-3-yl]diazenyl}benzoic acid (7). The reduced aniline for Baeyer-Mills reaction was obtained according to general procedure D using **19a** (112 mg, 0.328 mmol, 1.0 equiv.) and Pd/C (10 wt%, 59.3 mg, 0.056 mmol, 0.17 equiv.) to give a pale-orange crude oil (106 mg, quantitative) which was directly used (32.0 mg, 0.10 mmol, 1.0 equiv.) for the preparation of **7** according to general procedure E further using 4-amino-2-hydroxybenzoic acid (**20b**, 100 mg, 0.653 mmol, 2.0 equiv.) and Oxone® (805 mg, 1.31 mmol, 4.0 equiv.) to give **7** as an orange solid (32 mg, 14% over three steps). ^{1}H NMR (500 MHz, DMSO-*d*$_6$): δ 7.89 (d, *J* = 8.2 Hz, 1H), 7.82 (dd, *J* = 8.1, 2.1 Hz, 1H), 7.57 (d, *J* = 2.1 Hz, 1H), 7.52 (d, *J* = 8.2 Hz, 1H), 7.34 (d, *J* = 1.8 Hz, 1H), 7.26 (dd, *J* = 7.9, 1.8 Hz, 1H), 7.21 (dd, *J* = 8.2, 1.9 Hz, 1H), 7.15 (d, *J* = 1.9 Hz, 1H), 7.05 (d, *J* = 7.9 Hz, 1H), 4.54 (s, 1H), 2.47-2.23 (m, 2H), 2.07 (s, 3H), 1.83-1.69 (m, 4H), 1.37 (h, *J* = 7.4 Hz, 2H), 0.74-0.67 (m, 9H) ppm. ^{13}C NMR (126 MHz, DMSO-*d*$_6$): δ 171.3, 161.8, 156.1, 149.8, 146.2, 142.2, 140.9, 138.2, 136.7, 131.5, 131.0, 128.4, 126.6, 123.2, 123.1, 122.4, 115.3, 113.0, 110.6, 75.5, 34.7, 34.7, 23.4, 19.8, 13.6, 8.1 ppm.HRMS (ESI): *m/z* calculated 461.2435 for $C_{28}H_{32}O_4N_2$+H^+, found 461.2415 $[M+H]^+$.

(*E*)-2-Hydroxy-4-{[4'-(3-hydroxypentan-3-yl)-2-methyl-2'-propyl[1,1'-biphenyl]-4-yl]diazenyl}benzoic acid (8). Synthesis was performed according to general procedure E using a solution of **31a** (50.2 mg, 0.328 mmol, 2.0 equiv.) in CH_2Cl_2 (5.5 mL, 0.06 M), a solution of Oxone® (605 mg, 0.984 mmol, 6.0 equiv.) in H_2O (2.8 mL, 0.35 M) and **29** (51.1 mg, 0.164 mmol, 1.0 equiv.) to give to **8** as an orange solid (6.0 mg, 8.0 %). ^{1}H NMR (500 MHz, DMSO-*d*$_6$): δ 7.90 (d, *J* = 8.1 Hz, 1H), 7.82 (d, *J* = 2.1

Hz, 1H), 7.74 (dd, *J* = 8.1, 2.1 Hz, 1H), 7.36 – 7.29 (m, 2H), 7.26 (dd, *J* = 7.7, 1.9 Hz, 1H), 7.20 (dd, *J* = 8.1, 1.9 Hz, 1H), 7.15 (d, *J* = 1.9 Hz, 1H), 7.03 (d, *J* = 7.8 Hz, 1H), 4.55 (s, 1H), 2.47-2.23 (m, 2H), 2.10 (s, 3H), 1.84-1.67 (m, 4H), 1.37 (q, *J* = 7.4 Hz, 2H), 0.79-0.61 (m, 9H) ppm. ^{13}C NMR (126 MHz, acetone-d_6): δ 175.4, 162.2, 156.0, 151.6, 146.0, 145.3, 138.9, 137.6, 137.3, 132.5, 130.6, 128.4, 126.8, 124.3, 123.2, 119.9, 119.6, 112.5, 110.2, 76.2, 48.9, 35.2, 23.9, 19.4, 13.2, 7.5 ppm. HRMS (ESI): *m/z* calculated 461.2435 for $C_{28}H_{32}O_4N_2$+H$^+$, found 461.2471 [M+H]$^+$.

(*E*)-5-{[4'-(3-Hydroxypentan-3-yl)-2-methyl-2'-propyl[1,1'-biphenyl]-4-yl]diazenyl}-1,3-dihydro-2*H*-benzo[d]imidazol-2-one (9). **28** (150 mg, 0.439 mmol, 1.0 equiv.) and 5-amino-1,3-dihydro-2*H*-benzo[d]imidazol-2-one (**31b**, 164 mg, 1.10 mmol, 2.5 equiv.) were dissolved in ethanol (1.5 mL, 0.29 M) in a dry 10 mL microwave reactor vessel, and an aqueous solution (0.4 mL, 10.0 M) of KOH (246 mg, 4.39 mmol, 10.0 equiv.) was added to the mixture. The reaction mixture was placed in a microwave reactor and exposed to microwave irradiation at 120 °C for 30 minutes. The reaction mixture was quenched with water (5.0 mL) and extracted with EtOAc (3x10 mL), the combined organic layers were washed with water (1×15 mL) and brine (1x15 mL), dried over Na_2SO_4, filtered and concentrated under reduced pressure. The crude product was purified by aFCC using 30%-70% EtOAc in cyclohexane as gradient to obtain **9** as a yellow solid (13 mg, 6.3%). ^{1}H NMR (400 MHz, acetone-d_6): δ 9.97 (s, 1H), 9.89 (s, 1H), 7.84 (d, *J* = 2.0 Hz, 1H), 7.81-7.74 (m, 2H), 7.66 (d, *J* = 1.8 Hz, 1H), 7.46 (d, *J* = 1.9 Hz, 1H), 7.38-7.29 (m, 2H), 7.23 (d, *J* = 8.2 Hz, 1H), 7.08 (d, *J* = 7.9 Hz, 1H), 3.62 (s, 1H), 2.57-2.33 (m, 2H), 2.17 (s, 3H), 1.97-1.82 (m, 4H), 1.47 (h, *J* = 7.6 Hz, 2H), 0.85-0.74 (m, 9H) ppm. ^{13}C NMR (101 MHz, acetone-d_6): δ 170.0, 165.3, 151.7, 147.8, 146.0, 144.2, 139.0, 137.8, 137.1, 133.2, 130.6, 128.5, 126.8, 123.9, 123.2, 120.9, 119.4, 108.5, 99.9, 76.2, 59.6, 35.2, 23.9, 19.4, 13.2, 7.8 ppm. HRMS (ESI): m/z calculated 457.2598 for $C_{28}H_{32}N_4O_2$+H$^+$, found 457.2564 ([M+H]$^+$).

(*E*)-6-{[4'-(3-Hydroxypentan-3-yl)-2-methyl-2'-propyl[1,1'-biphenyl]-4-yl]diazenyl}-2-naphthoic acid (10). **32** (35 mg, 0.069 mmol, 1.0 equiv.) and LiOH (19 mg, 0.34 mmol, 5.0 equiv.) were dissolved in a mixture of THF and H_2O (0.05 M, 1:1) and stirred at 45 ℃ for 4 h. After cooling to r.t., the mixture was diluted with H_2O (10 mL) and the pH was adjusted to 3-4 using aqueous HCl solution (2 M). The mixture was extracted with EtOAc (3×10 mL) and the combined organic layers were washed with water (1×15 mL) and brine (1×15 mL), dried over Na_2SO_4, filtered and concentrated under reduced pressure. The crude product was purified by flash chromatography (SiO_2, gradient: 0%-10 % MeOH in CH_2Cl_2) to give **10** as an orange solid (15 mg, 44%). ^{1}H NMR (500 MHz, acetone-d_6): δ 11.50 (brs, 1H), 8.75 (s, 1H), 8.65-8.61 (m, 1H), 8.27 (dd, *J* = 8.7, 4.3 Hz, 2H), 8.21-8.16 (m, 2H), 7.96 (d, *J* = 2.0 Hz, 1H), 7.90 (dd, *J* = 8.0, 2.1 Hz, 1H), 7.46 (d, *J* = 1.9 Hz, 1H), 7.40-7.34 (m, 2H), 7.09 (d, *J* = 7.9 Hz, 1H), 3.61 (s, 1H), 2.58 -2.31 (m, 2H), 2.19 (s, 3H), 1.97-1.89 (m, 2H), 1.89-1.81 (m, 2H), 1.51-1.42 (m, 2H), 0.82-0.76 (m, 9H) ppm. ^{13}C NMR (126 MHz, acetone-d_6): δ 167.4, 152.7, 152.6, 147.0, 146.4, 139.8, 138.5, 138.4, 136.8, 134.9, 131.7, 131.7, 131.6, 130.5, 130.3, 129.3, 128.0, 127.7, 127.3, 125.3, 124.2, 120.7, 118.6, 77.1, 36.1, 24.8, 20.3, 14.2, 8.4 ppm. HRMS (ESI): m/z calculated 493.2497 for $C_{32}H_{34}N_2O_3$-H$^+$, found 493.2494 ([M-H]$^-$).

Methyl 4-bromo-3-(bromomethyl)benzoate (12)[3]. A 50 mL round bottom flask was charged with methyl 4-bromo-3-methylbenzoate (**11**, 2.0 g, 8.7 mmol, 1.0 equiv.) in MeCN (20 mL, 0.44 M). *N*-bromosuccinimide (NBS) (1.7 g, 9.6 mmol, 1.1 equiv.) and benzoic peroxyanhydride ($(PhCO)_2$) (0.42 g, 1.7 mmol, 0.2 equiv.) were added. The mixture was stirred at 80 °C for 16 h. The clear yellow solution was concentrated under reduced pressure, and the resulting residue was purified by flash-chromatographed to give **12** as colorless crystalline solid (1.1 g, 42%). ^{1}H NMR (600 MHz, $CDCl_3$): δ 8.13-8.11 (m, 1H), 7.83-7.80 (m, 1H), 7.66 (d, *J* = 8.4 Hz, 1H), 4.62 (s, 2H), 3.93 (s, 3H) ppm. LC-MS (ESI): *m/z* 239.1 ($[M-H_2O+H]^+$).

3-(4-Bromo-3-methylphenyl)pentan-3-ol (13)[4]. Synthesis was performed according to general procedure B using compound **11** (500 mg, 2.18 mmol, 1.0 equiv.), anhydrous THF (2.5 mL, 0.87 M) and a solution of EtMgBr (1.0 M in THF, 5.46 mL, 5.46 mmol, 2.5 equiv.) to give **13** as a pale-yellow oil (440 mg, 78%). ^{1}H NMR (400 MHz, $CDCl_3$): δ 7.46 (d, *J* = 8.3 Hz, 1H), 7.25 (d, *J* = 2.6 Hz, 1H), 7.02 (dd, *J* = 8.4, 2.4 Hz, 1H), 2.40 (s, 3H), 1.89-1.71 (m, 4H), 0.75 (t, *J* = 7.4 Hz, 6H) ppm. LC-MS (ESI): *m/z* 241.1 ($[M-H_2O+H]^+$).

3-[4-Bromo-3-(bromomethyl)phenyl]pentan-3-ol (14). A 100 mL round bottom flask was charged with 3-(4-bromo-3-methylphenyl)pentan-3-ol (**13**, 890 mg, 3.46 mmol, 1.0 equiv.) in MeCN (60 mL). NBS (678 mg, 3.81 mmol, 1.1 equiv.) and $(PhCOO)_2$ (168 mg, 0.692 mmol, 0.2 equiv.) were added to the solution. The resulting yellow mixture was stirred at 80 °C for 18 h. After cooling to r.t., water (50 mL) was added, the mixture was extracted with CH_2Cl_2 (3×40 mL), the combined organic layers were washed with brine (50 mL), dried over Na_2SO_4, filtered, and concentrated. The crude residue was purified using flash chromatography (SiO_2; 20% EtOAc in hexane mixture) to yield **14** as a yellow oil (925 mg, 79%). ^{1}H NMR (600 MHz, $CDCl_3$): δ 7.52 (d, *J* = 8.4 Hz, 1H), 7.49 (d, *J* = 2.3 Hz, 1H), 7.16 (dd, *J* = 8.4, 2.4 Hz, 1H), 4.61 (s, 2H), 1.91-1.70 (m, 5H), 0.76 (t, *J* = 7.4 Hz, 6H) ppm. LC-MS (ESI): *m/z* 337.3 ($[M+H]^+$).

3-(4-Bromo-3-propylphenyl)pentan-3-ol (16)[4]. Synthesis was performed according to general procedure B using **12** (500 mg, 1.62 mmol, 1.0 equiv.), anhydrous THF (2.5 mL, 0.65 M) and a solution of EtMgBr (1.0 M in THF, 5.68 mL, 5.68 mmol, 3.5 equiv) to give **16** as a colorless oil (130 mg, 28%). ^{1}H NMR (400 MHz, $CDCl_3$): δ 7.46 (d, *J* = 8.3 Hz, 1H), 7.22 (d, *J* = 2.4 Hz, 1H), 7.03 (dd, *J* = 8.3, 2.4 Hz, 1H), 2.74-2.68 (m, 2H), 1.89-1.72 (m, 4H), 1.69-1.59 (m, 2H), 0.97 (t, *J* = 7.3 Hz, 3H), 0.75 (t, *J* = 7.4 Hz, 6H) ppm. LC-MS (ESI): *m/z* 267.1 ($[M-H_2O+H]^+$).

3-[4-Bromo-3-(but-3-en-1-yl)phenyl]pentan-3-ol (17). A 25 mL flame dried Schlenk flask was charged with **14** (804 mg, 2.38 mmol, 1.0 equiv.) in dry THF (4.0 mL) under N_2. Allylmagnesium bromide (**15**; 1 M solution in THF, 6 mL, 5.95 mmol, 2.5 equiv.) was added slowly via syringe at 0 °C. The mixture was allowed to warm to r.t. and was stirred for 16 h. The reaction was quenched by the slow addition of ice/water. Saturated aqueous NH_4Cl solution was added, the layers were separated, and the aqueous layer was further extracted with Et_2O (3×10 mL). The combined organic layers were dried over Na_2SO_4, filtered, and concentrated under reduced pressure. The resulting crude oil was purified using flash chromatography (SiO_2; 10% EtOAc in hexanes mixture) to give **17** as colorless oil (576 mg, 81%). ^{1}H NMR (600 MHz, $CDCl_3$): δ 7.45-7.48 (m, 1H), 7.23-7.21 (m, 1H), 7.06-7.03 (m, 1H), 5.91-5.82 (m, 1H), 5.06-5.01

(m, 1H), 5.00-4.96 (m, 1H), 2.86-2.81 (m, 2H), 2.42-2.34 (m, 2H), 1.87-1.73 (m, 5H), 0.78-0.71 (m, 6H) ppm. LC-MS (ESI): *m/z* 279.2 ($[M-H_2O+H]^+$).

3-(2'-Methyl-5'-nitro-2-propyl[1,1'-biphenyl]-4-yl)pentan-3-ol (19a). Synthesis was performed according to general procedure C using **16** (116 mg, 0.405 mmol, 1.0 equiv.), **18** (80.5 mg, 0.445 mmol, 1.1 equiv.), Pd(dppf)Cl_2·CH_2Cl_2 (14.2 mg, 0.0174 mmol, 0.043 equiv.) and K_3PO_4 (283 mg, 1.34 mmol, 3.3 equiv) to give **19a** as a colorless oil (54.3 mg, 39%). ^{1}H NMR (500 MHz, $CDCl_3$): δ 8.14 (d, *J* = 2.4 Hz, 1H), 8.07 (dd, *J* = 8.3, 2.5 Hz, 1H), 7.33-7.29 (m, 2H), 7.24 (dd, *J* = 7.9, 2.0 Hz, 1H), 6.99 (d, *J* = 7.9 Hz, 1H), 2.40 (dt, *J* = 14.6, 7.6 Hz, 1H), 2.23 (dt, *J* = 13.7, 7.8 Hz, 1H), 2.14 (s, 3H), 1.95-1.81 (m, 4H), 1.46-1.35 (m, 2H), 0.81 (t, *J* = 7.4 Hz, 7H), 0.76 (t, *J* = 7.3 Hz, 3H) ppm. LC-MS(ESI): *m/z* 324.2 ($[M-H_2O+H]^+$).

3-(2,2'-Dimethyl-5'-nitro[1,1'-biphenyl]-4-yl)pentan-3-ol (19b). Synthesis was performed according to general procedure C using **13** (155 mg, 0.603 mmol, 1.0 equiv.), **18** (120 mg, 0.663 mmol, 1.1 equiv.), Pd(dppf)Cl_2·CH_2Cl_2 (21.2 mg, 0.0260 mmol, 0.043 equiv.) and K_3PO_4 (422 mg, 1.99 mmol, 3.3 equiv) to give **19b** as a colorless oil (157 mg, 83%). ^{1}H NMR (400 MHz, $CDCl_3$): δ 8.11 (dd, *J* = 8.4, 2.5 Hz, 1H), 8.02 (d, *J* = 2.5 Hz, 1H), 7.42 (d, *J* = 8.4 Hz, 1H), 7.32 (d, *J* = 1.9 Hz, 1H), 7.24 (dd, *J* = 7.9, 1.9 Hz, 1H), 7.04 (d, *J* = 7.9 Hz, 1H), 2.15 (s, 3H), 2.06 (s, 3H), 1.96-1.80 (m, 4H), 0.82 (t, *J* = 7.4 Hz, 6H) ppm. LC-MS (ESI): *m/z* 296.1 ($[M-H_2O+H]^+$).

3-[2-(But-3-en-1-yl)-2'-methyl-5'-nitro[1,1'-biphenyl]-4-yl]pentan-3-ol (19c). A flame-dried 10 mL Schlenk flask under N_2, was charged with **17** (150 mg, 0.505 mmol, 1.0 equiv.), **18** (110 mg, 0.606 mmol, 1.2 equiv.), Pd(PPh_3)$_4$ (29.2 mg, 0.0253 mmol, 0.05 equiv.). Degassed and dry 1,4-dioxane (2.5 mL) was added followed by a degassed aq. Na_2CO_3 solution (1 M, 1.26 mL, 2.5 equiv.) and MeOH (21.0 µL, 0.505 mmol, 1 equiv.). The mixture was stirred at 90 °C for 24 h. H_2O (5 mL) was added, and the mixture was extracted with CH_2Cl_2 (3 x 20 mL). The combined organic layers were dried over Na_2SO_4, filtered, and concentrated under pressure. The resulting crude oil was purified using flash chromatography (SiO_2; 20% EtOAc in hexane) to give **19c** as a yellow oil (98 mg, 55%). ^{1}H NMR (600 MHz, $CDCl_3$): δ 8.12 (dd, *J* = 8.4, 2.5 Hz, 1H), 8.04 (d, *J* = 2.5 Hz, 1H), 7.41 (d, *J* = 8.4 Hz, 1H), 7.33 (d, *J* = 1.9 Hz, 1H), 7.26-7.24 (m, 1H), 7.03 (d, *J* = 7.9 Hz, 1H), 5.63 (ddt, *J* = 17.0, 10.4, 6.7 Hz, 1H), 4.90-4.82 (m, 2H), 2.58-2.48 (m, 1H), 2.41-2.33 (m, 1H), 2.18 (s, 1H), 2.17-2.13 (m, 5H), 1.95-1.81 (m, 4H), 0.81 (t, *J* = 7.4 Hz, 6H). LC-MS (ESI): *m/z* 336.5 ($[M-H_2O+H]^+$).

Dimethyl (*E*)-4-{[4'-(3-hydroxypentan-3-yl)-6-methyl-2'-propyl[1,1'-biphenyl]-3-yl] diazenyl}phthalate (21a). The reduced aniline for Baeyer-Mills reaction was obtained according to general procedure D using **19a** (54.3 mg, 0.159 mmol, 1.0 equiv.) and Pd/C (10 wt%, 28.4 mg, 0.0270 mmol, 0.17 equiv.) to give the crude product as an oil (27.7 mg, 56%, 0.0890 mmol) which was used without further purification in the preparation of **21a** according to general procedure E further using dimethyl 4-aminophthalate (**20a**, 27.9 mg, 0.133 mmol, 1.5 equiv.) and Oxone® (246 mg, 0.400 mmol, 4.5 equiv.) to give **21a** as an orange oil (30 mg, 14% over three steps). ^{1}H NMR (400 MHz, $CDCl_3$): δ 8.21 (d, *J* = 1.9 Hz, 1H), 8.03 (dd, *J* = 8.2, 2.0 Hz, 1H), 7.89-7.84 (m, 2H), 7.77 (d, *J* = 2.1 Hz, 1H), 7.42 (d, *J* = 8.2 Hz,

1H), 7.31 (d, *J* = 2.0 Hz, 1H), 7.23 (dd, *J* = 8.0, 2.0 Hz, 1H), 7.08 (d, *J* = 7.9 Hz, 1H), 3.94 (s, 3H), 3.94 (s, 3H), 2.40 (ddt, *J* = 55.4, 13.7, 7.7 Hz, 2H), 2.14 (s, 3H), 1.95-1.80 (m, 4H), 1.50-1.39 (m, 2H), 0.82 (t, *J* = 7.4 Hz, 6H), 0.77 (t, *J* = 7.3 Hz, 3H) ppm. HRMS (ESI): *m/z* calculated 517.2697 for $C_{31}H_{36}N_2O_5+H^+$, found: 517.2691 ($[M+H]^+$).

Dimethyl (*E*)-4-[(4'-(3-hydroxypentan-3-yl)-2',6-dimethyl[1,1'-biphenyl]-3-yl)diazenyl]phthalate (21b). The reduced aniline for Baeyer-Mills reaction was obtained according to general procedure D using **19b** (78.7 mg, 0.251 mmol, 1.0 equiv.) and Pd/C (10 wt%, 44.9 mg, 0.0427 mmol, 0.17 equiv.) to give the crude product as an oil (47.6 mg, 67%, 0.168 mmol) that was used without further purification in the preparation of **21b** according to general procedure E further using **20a** (52.7 mg, 0.252 mmol, 1.5 equiv.) and Oxone® (465 mg, 0.756 mmol, 4.5 equiv.) to give **21b** as an orange solid (61.1 mg, 50% over three steps). ^1H NMR (400 MHz, $CDCl_3$): δ 8.21 (d, *J* = 1.9 Hz, 1H), 8.03 (dd, *J* = 8.3, 2.0 Hz, 1H), 7.89-7.84 (m, 2H), 7.75 (d, *J* = 2.1 Hz, 1H), 7.43 (d, *J* = 8.2 Hz, 1H), 7.31 (d, *J* = 1.9 Hz, 1H), 7.23 (dd, *J* = 7.7, 1.9 Hz, 1H), 7.10 (d, *J* = 7.9 Hz, 1H), 3.94 (s, 3H), 3.94 (s, 3H), 2.14 (s, 3H), 2.11 (s, 3H), 1.93-1.81 (m, 4H), 0.83 (t, *J* = 7.4 Hz, 6H) ppm. HRMS (ESI): *m/z* calculated 489.2384 for $C_{29}H_{32}N_2O_5+H^+$, found: 489.2381 ($[M+H]^+$).

Dimethyl (*E*)-4-{[2'-(butyl)-4'-(3-hydroxypentan-3-yl)-6-methyl[1,1'-biphenyl]-3-yl]diazenyl}phthalate (21c). The reduced aniline for Baeyer-Mills reaction was obtained according to general procedure D using **19c** (60.0 mg, 0.170 mmol, 1.0 equiv.) and Pd/C (10 wt%, 30.4 mg, 0.0289 mmol, 0.17 equiv.) to give the crude product as a pale-yellow oil (53 mg, 96%, 0.163 mmol) which was used without further purification in the preparation of **21c** according to general procedure E further using **20a** (57.8 mg, 0.276 mmol, 2.0 equiv.) and Oxone® (602 mg, 0.979 mmol, 6.0 equiv.) to give **21c** as an orange solid (53 mg, 72% over three steps). ^1H NMR (600 MHz, CD_3CN): δ 8.15 (d, *J* = 2.0 Hz, 1H), 8.07 (dd, *J* = 8.2, 2.0 Hz, 1H), 7.90-7.86 (m, 2H), 7.69 (dd, *J* = 11.7, 2.2 Hz, 1H), 7.53-7.50 (m, 1H), 7.37-7.34 (m, 1H), 7.28-7.24 (m, 1H), 7.10-7.07 (m, 1H), 3.89-3.87 (m, 6H), 2.92 (s, 1H), 2.53-2.46 (m, 2H), 2.12 (s, 3H), 1.89-1.75 (m, 4H), 1.38 (p, *J* = 7.6 Hz, 2H), 1.18-1.09 (m, 2H), 0.78-0.69 (m, 9H) ppm. LC-MS (ESI): *m/z* 513.5 ($[M-H_2O+H]^+$).

1-(4-Hydroxy-3-propylphenyl)propan-1-one (24)[5]. Propionyl chloride (**23**, 408 mg, 4.4 mmol, 1.2 equiv.) was added to 2-propylphenol (**22**, 500 mg, 3.67 mmol, 1.0 equiv.) in an oven-dried 10 mL Schlenk flask under Ar. The pale-yellow mixture was cooled in an ice-water bath to 0 °C and trifluoromethanesulfonic acid (CF_3SO_3H) (281 mg, 1.84 mmol, 98 %, 0.5 equiv.) was added dropwise. The mixture was allowed to warm to r.t. and stirred overnight. The reaction was quenched at 0 °C by the addition of H_2O (15 mL). The mixture was extracted with EtOAc (3×10 mL), the combined organic layers were washed with H_2O (2× 15 mL) and brine (1x 15 mL), dried over Na_2SO_4, filtered and concentrated under reduced pressure. The resulting residue was purified by aFCC using 3%-8% EtOAc in cyclohexane as gradient to obtain **24** as a yellow solid (316 mg, 45%). ^1H NMR (400 MHz, acetone-d_6): δ 8.96 (s, 1H), 7.79 (d, *J* = 2.3 Hz, 1H), 7.72 (dd, *J* = 8.4, 2.3 Hz, 1H), 6.90 (d, *J* = 8.4 Hz, 1H), 2.94 (q, *J* = 7.3 Hz, 2H), 2.67-2.60 (m, 2H), 1.70-1.59 (m, 2H), 1.11 (t, *J* = 7.3 Hz, 3H), 0.95 (t, *J* = 7.4 Hz, 3H) ppm. MS (APCI+): *m/z* 193.1 ($[M + H]^+$).

4-Propionyl-2-propylphenyl trifluoromethanesulfonate (25)[6]. In an oven-dried 25 mL Schlenk flask under Ar, **24** (534 mg, 2.78 mmol, 1.0 equiv.) was dissolved in anhydrous toluene (5.6 mL, 0.5 M) and triethylamine (TEA, 844 mg, 8.34 mmol, 3.0 equiv.) was added. The mixture was cooled to 0 °C in an ice-water bath and then trifluoromethanesulfonic anhydride $(CF_3SO_2)_2O$ (1.02 g, 3.61 mmol, 1.3 equiv.) was added dropwise. The mixture was allowed to warm to r.t. and stirred for 3h. The solvent was removed under reduced pressure and the crude product was purified by aFCC using 2%-6% EtOAc in cyclohexane as gradient to give **25** as a yellow oil (695 mg, 77%). ^{1}H NMR (400 MHz, acetone): δ 8.09 (d, *J* = 2.3 Hz, 1H), 8.01 (dd, *J* = 8.6, 2.3 Hz, 1H), 7.51 (d, *J* = 8.6 Hz, 1H), 3.10 (q, *J* = 7.2 Hz, 2H), 2.80-2.75 (m, 2H), 1.80-1.63 (m, 2H), 1.15 (t, *J* = 7.2 Hz, 3H), 0.99 (t, *J* = 7.3 Hz, 3H) ppm. MS (APCI+): *m/z* 325.2 ($[M + H]^+$).

4-(3-Hydroxypentan-3-yl)-2-propylphenyl trifluoromethanesulfonate (26)[6]. Synthesis was performed according to general procedure B using **25** (756 mg, 2.33 mmol, 1.0 equiv.), anhydrous THF (4.7 mL, 0.5 M) and a solution of EtMgBr (1.0 M in THF, 3.03 mL, 1.3 equiv.) to give **26** as a pale-yellow oil (531 mg, 64%). ^{1}H NMR (400 MHz, acetone-*d*$_6$): δ 7.53 (d, *J* = 2.4 Hz, 1H), 7.44 (dd, *J* = 8.7, 2.4 Hz, 1H), 7.29 (d, *J* = 8.6 Hz, 1H), 3.77 (s, 1H), 2.74-2.69 (m, 2H), 1.91-1.74 (m, 4H), 1.73-1.62 (m, 1H), 0.96 (t, *J* = 7.3 Hz, 3H), 0.72 (t, *J* = 7.4 Hz, 6H) ppm. MS (APCI+): *m/z* 337.2 ($[M-H_2O+H]^+$).

3-(2'-Methyl-4'-nitro-2-propyl[1,1'-biphenyl]-4-yl)pentan-3-ol (28). **26** (698 mg, 1.97 mmol, 1.0 equiv.), 2-methyl-4-nitrophenylboronic acid (**27**, 446 mg, 2.36 mmol, 1.2 equiv., 96%), K_2CO_3 (681 mg, 4.93 mmol, 2.5 equiv.), toluene (10 mL, 0.2 M), ethanol (2.0 mL, 1.0 M) and H_2O (1.3 mL, 1.0 M) were added into a 50 mL Schlenk flask under Ar, and XPhos Pd G2 (30.7 mg, 0.197 mmol, 0.02 equiv.) was added. The mixture was stirred at 85 °C for 1 hour. After cooling to r.t., the mixture was filtered through a pad of celite, washed with EtOAc, the filtrate was dried over Na_2SO_4, filtered and concentrated under pressure with the rotary evaporator. The resulting residue was purified by aFCC using 0%-10% EtOAc in cyclohexane as gradient and RP-CC using a gradient of H_2O with 25% - 100% MeCN to give **28** as a yellow oil (619 mg, 92%). ^{1}H NMR (400 MHz, acetone-*d*$_6$): δ 8.19 (d, *J* = 2.4 Hz, 1H), 8.14-8.09 (m, 1H), 7.46 (d, *J* = 1.9 Hz, 1H), 7.42 (d, *J* = 8.4 Hz, 1H), 7.36 (dd, *J* = 7.9, 1.9 Hz, 1H), 7.04 (d, *J* = 7.9 Hz, 1H), 3.63 (s, 1H), 2.51-2.43 (m, 1H), 2.32-2.24 (m, 1H), 1.96-1.79 (m, 4H), 1.47-1.37 (m, 2H), 0.81-0.73 (m, 9H) ppm. MS (APCI+): *m/z* 342.3 ($[M+H]^+$).

3-(4'-Amino-2'-methyl-2-propyl[1,1'-biphenyl]-4-yl)pentan-3-ol (29). MeOH (10 mL), H_2O (5.0 mL), zinc (467 mg, 7.15 mmol, 5.0 equiv.) and NH_4Cl (382 mg, 7.15 mmol, 5.0 equiv.) were added to a solution of **28** (488 mg, 1.43 mmol, 1.0 equiv.) in THF (20 mL) under Ar. The reaction mixture was stirred at r.t. for 15 min and then heated to reflux for 6 h. After cooling to r.t., the mixture was filtered through a pad of celite, washed with EtOAc, the filtrate was dried over Na_2SO_4, filtered and concentrated under reduced pressure. The resulting residue was purified by aFCC using 8%-25% EtOAc in cyclohexane as gradient to give **29** as a colorless solid (411 mg, 92%). ^{1}H NMR (400 MHz, acetone-*d*$_6$): δ 7.34 (d, *J* = 1.9 Hz, 1H), 7.23 (dd, *J* = 7.9, 2.0 Hz, 1H), 6.96 (d, *J* = 7.9 Hz, 1H), 6.79 (d, *J* = 8.0 Hz, 1H), 6.59 (d, *J* = 2.4 Hz, 1H), 6.55-6.51

(m, 1H), 4.50 (s, 2H), 3.50 (s, 1H), 2.50-2.42 (m, 1H), 2.37-2.29 (m, 1H), 1.90-1.76 (m, 7H), 1.46-1.37 (m, 2H), 0.79-0.73 (m, 9H) ppm. MS (APCI+): *m/z* 312.3 ($[M+H]^+$).

3-(2'-Methyl-4'-nitroso-2-propyl[1,1'-biphenyl]-4-yl)pentan-3-ol (30). A solution of Oxone® (3.28 g, 5.33 mmol, 6.0 equiv.) in H_2O (15.2 mL, 0.35 M) was added to a solution of **29** (277 mg, 0.889 mmol, 1.0 equiv.) in CH_2Cl_2 (14.8 mL, 0.06 M). The resulting biphasic mixture was stirred vigorously at r.t. for 18 h. The phases were separated, and the organic layer was washed with 1 M HCl (1×10 mL), saturated aq. $NaHCO_3$ solution (1×10 mL), H_2O (2×15 mL) and brine (1×15 mL), dried over Na_2SO_4, filtered and concentrated under reduced pressure. The resulting residue was purified by aFCC using 5%-20% EtOAc in cyclohexane as gradient to give **30** as a green oil (140 mg, 48%). ^1H NMR (400 MHz, $CDCl_3$): δ 7.83-7.79 (m, 1H), 7.78-7.73 (m, 1H), 7.39 (d, *J* = 7.9 Hz, 1H), 7.32 (d, *J* = 1.9 Hz, 1H), 7.24 (dd, *J* = 7.9, 1.9 Hz, 1H), 7.01 (d, *J* = 7.9 Hz, 1H), 2.47-2.39 (m, 1H), 2.31-2.22 (m, 1H), 2.19 (s, 3H), 1.96-1.80 (m, 4H), 1.66 (s, 1H), 1.48-1.36 (m, 2H), 0.85-0.79 (m, 6H), 0.76 (t, *J* = 7.4 Hz, 3H) ppm. MS (APCI+): *m/z* 325.7 ($[M+H]^+$).

Methyl *(E)*-6-{[4'-(3-hydroxypentan-3-yl)-2-methyl-2'-propyl[1,1'-biphenyl]-4-yl] diazenyl}-2-naphthoate (32). Methyl 6-amino-2-naphthoate (**31c**, 223 mg, 1.11 mmol, 3.0 equiv.) and KOH (104 mg, 1.85 mmol, 5.0 equiv.) were added to an oven-dried Schlenk flask under Ar. A solution of **30** (120 mg, 0.369 mmol, 1.0 equiv.) in anhydrous DMF (3.7 mL, 0.1 M) was added dropwise at r.t while stirring vigorously. The mixture was stirred for another 10 min with its color turning dark. The mixture was then poured into an ice/water-cooled aq. HCl solution (0.5 M, 20 mL) resulting in an orange precipitate which was filtered off and washed with cold water until the filtrate was neutral. The raw product was purified by column chromatography on silica using 0%-20 % EtOAc in cyclohexane as gradient to give **32** as an orange solid (42 mg, 22%). ^1H NMR (400 MHz, $CDCl_3$): δ 8.69-8.63 (m, 1H), 8.50-8.46 (m, 1H), 8.17-8.12 (m, 2H), 8.09-8.03 (m, 2H), 7.90 (d, *J* = 2.0 Hz, 1H), 7.87-7.83 (m, 1H), 7.36-7.31 (m, 2H), 7.24 (dd, *J* = 7.9,2.0 Hz, 1H), 7.08 (d, *J* = 7.9 Hz, 1H), 4.01 (s, 3H), 2.53-2.43 (m, 1H), 2.38-2.29 (m, 1H), 2.17 (s, 3H), 1.95-1.82 (m, 4H), 1.69 (s, 1H), 1.50-1.41 (m, 2H), 0.85-0.76 (m, 9H) ppm. MS (APCI+): *m/z* 508.6 ($[M+H]^+$).

Photophysical characterization

UV–vis spectra were recorded using a Varian Cary 60 Bio UV–visible spectrophotometer with BRAND Ultra-Micro UV-Cuvettes (10 mm light path). Experiments were carried out at r.t.. Switching was achieved using light-emitting diode (LED) light sources at appropriate wavelengths. The LEDs were pointed directly into the top of the sample cuvette. An initial spectrum of the photohormones (25 µM in DMSO) was recorded (dark-adapted state) and then again following illumination at λ = 365 nm for 1 min (cis-adapted state). A third spectrum was recorded after irradiation at λ = 465 nm for 1 min (trans-adapted state). For the photohormone **10**, spectra were recorded at different irradiation wavelengths (280–550 nm) starting from no irradiation. To observe reversible trans ← → cis isomerization of **10** (25 µM in DMSO), absorption at λ_{abs} = 351 nm was constantly measured (using kinetics mode, measuring every 0.5 s), while alternating illumination at λ = 365 or 465 nm over 10 min (30 s per cycle). To observe thermal relaxation, a sample of **10** (6.1 mM in acetone) in a clear NMR tube was first irradiated with 365 nm for 30 min for complete switching into the (*Z*)-form and then kept in the dark with repeated recording of ^{1}H NMR spectra (500 MHz) over 96 h to determine (*E*)/(*Z*)-ratios over time. Photostationary states (PSS) of **10** after irradiation were determined by irradiating the sample at λ = 365 nm for 5 min, and at λ = 465 nm for 15 min with subsequent determination of (*E*)/(*Z*)-ratios via ^{1}H NMR.

In vitro assays

Gal4-hybrid reporter gene assays. Reporter gene assays were performed in HEK293T cells (German Collection of Microorganisms and Cell Culture GmbH, DSMZ) as reported previously using Gal4-NR LBD fusion receptor plasmids each coding for the hinge region and LBD of the canonical isoform of the respective human nuclear receptor.[7,8] pFR-Luc (Stratagene, La Jolla, CA, USA) served as a reporter plasmid and pRL-SV40 (Promega, Madison, WI, USA) was used for normalization of transfection efficiency and test compound toxicity. Cells were cultured in Dulbecco's modified Eagle's medium (DMEM), high glucose supplemented with 10% fetal calf serum (FCS), sodium pyruvate (1 mM), penicillin (100 U/mL), and streptomycin (100 μg/mL) at 37 °C and 5% CO_2, and seeded in 96-well plates (3 × 10^4 cells/well). After 24 h, the medium was changed to Opti-MEM without supplements, and cells were transiently transfected using Lipofectamine LTX reagent (Invitrogen, Carlsbad, CA, USA) according to the manufacturer's protocol. Five hours after transfection, cells were incubated with the test compounds solubilized with 0.1% DMSO in Opti-MEM supplemented with penicillin (100 U/mL), streptomycin (100 μg/mL). 0.1% DMSO served as untreated control. For illumination during incubation, we performed pulsed irradiation with the indicated wavelength using a CellDisco[9] containing an array of 24 x 5 mm LEDs (LEDs were supplied by Roithner Lasertechnik), pulsed at 100 ms "on" every 10 s. After 14−16 h incubation, luciferase activity was measured using the Dual-Glo Luciferase Assay System (Promega) according to the manufacturer's protocol on a Tecan Spark luminometer (Tecan Deutschland GmbH, Crailsheim, Germany). Each concentration was tested in duplicates, and each experiment was performed independently at least three times. Firefly luciferase data were divided by renilla luciferase data and multiplied by 1000 to obtain relative light units (RLU). RLU data were normalized to the untreated control to obtain fold activation and fold activation data were normalized to the respective reference agonist to obtain relative activation. EC_{50} and IC_{50} values were calculated by fitting dose−response data with the equation "[agonist] vs response − variable slope (four parameters)" or "[inhibitor] vs response − variable slope (four parameters)" in GraphPad Prism (version 7, GraphPad software, La Jolla, CA, USA). All hybrid assays were validated with reference ligands and a reference ligand was contained on every plate to monitor assay performance.

Fluorescent Reporter Gene Assay. The fluorescence reporter gene assay was performed in HEK293T cells (DSMZ) using the Gal-fusion receptor plasmid pFA-CMV-VDR-LBD coding for the hinge region and LBD of the canonical human VDR isoform. The Gal4-responsive fluorescence reporter mCherry was expressed from plasmid pUAS-mCherry-NLS[10] (Addgene, entry 87695, Watertown, MA, USA). Cell culture, seeding and transfection were performed as described for Gal4-hybrid reporter gene assays. Five hours after transfection, the medium was changed to Opti-MEM supplemented with penicillin (100 U/mL) and streptomycin (100 μg/mL) and additionally containing 0.1% DMSO and **10** (1 μM), calcitriol (1 μM) or 0.1% DMSO alone as the untreated control. Each concentration was tested in duplicate, and each

experiment was repeated independently three times. CellDiscos[9] were used for pulsed irradiation at 370 nm or 450 nm to switch **10** between photostationary states. The live cells were assayed for fluorescence reporter intensity at multiple time points over 36 h. Fluorescence intensity (FI) was measured (λ_{ex} 585/10 nm, λ_{em} 610/10 nm) in bottom reading mode with a Tecan Spark Cyto (Tecan Deutschland GmbH). Fold FI was obtained by dividing the mean FI of a test sample by the mean FI of the untreated control of the respective measurement (time point).

***In vitro* osteogenesis model**. *Cell culture and osteogenesis*: Differentiation experiments of ASC52telo hTERT cells (ATCC® SCRC-4000™) were conducted according to a previously described procedure[11]. In brief, cells were grown in DMEM high glucose, supplemented with 10% fetal calf serum, sodium pyruvate (1 mM), penicillin (100 U/mL), and streptomycin (100 µg/mL) at 37 °C and 5% CO_2. Cells were seeded in standard culture medium at a density of 10,000 cells per well in 96-well plates. After adherence overnight, cells were incubated with osteogenic differentiation medium, composed of DMEM low glucose, supplemented with 10% fetal calf serum, L-glutamine (2 mM), penicillin (100 U/mL), streptomycin (100 µg/mL), dexamethasone (100 nM, #D4902, Merck KgaA, Darmstadt, Germany), 2-phospho-L-ascorbic acid (50 mM, CAS-No. 66170-10-3, Merck KGaA), glycerol 2-phosphate (10 mM, CAS-No. 13408-09-8, Fisher Scientific Inc., Germany), and DMSO (final concentration 0.1%) with or without the respective test compounds (**10**, calcitriol) at indicated concentrations. The cells were kept in differentiation medium for a total of 15 days with complete medium exchange every 48–72 h. For the undifferentiated control, ASC52telo hTERT cells were kept in standard culture medium alongside differentiating cells in the same 96-well plates for the whole duration of the experiment. All experiments were performed in the dark and under pulsed irradiation with a CellDisco[9] (370 nm; 100 ms/ 10 s). Data are derived from 6 independent experiments with 5–10 technical repeats each. *Alkaline phosphatase (ALP) activity*: After the 15-day differentiation procedure and test compound treatment, cells were washed with phosphate buffered saline (PBS) twice and incubated with *p*-nitrophenyl phosphate (10 mM, CAS-No. 52483-84-8, Merck KGaA) in a 100 mM TRIS-HCl buffer (pH 9.5) supplemented with 1 mM $MgCl_2$ and 0.1% Triton X-100 (150 µL per well). Absorbance was measured at 410 nm for 60 min every 5 min with an increase in absorbance reflecting phosphatase activity (nmol/min). For quantification via linear regression a *p*-nitrophenyl (CAS-No. 88-75-5, Merck KGaA) calibration curve was recorded. Changes in ALP activity are reported as fold-of-control compared to differentiated and DMSO treated cells. *Silvering according to von Kossa*: After the 15-day differentiation procedure and test compound treatment, cells were washed with PBS twice and fixed with formalin (10%, stabilized with methanol, 100 µL per well, #15071, Morphisto GmbH, Offenbach am Main, Germany) at r.t. for 15 min. The fixing solution was aspirated, and the fixed cells were washed three times with ultrapure water. Next, using a silvering kit according to von Kossa (#16764, Morphisto GmbH,), cells were incubated with 5% silver nitrate (1 h, r.t., under ambient light), 5% sodium carbonate formalin (30 min, r.t., under ambient light), and 5% sodium thiosulfate (15 min, r.t., under ambient light), each at 100 µL per well, respectively, with a washing step

(three times ultrapure water) between each incubation. Specimens were kept in water for subsequent analysis. For each well, 2–4 pictures were taken at a 4X magnification using a Motic®AE31E inverted microscope and a Moticam 1080 (Motic Hong Kong Ldt.). Images were corrected for skewed illumination, unspecific precipitates were identified and removed, and the brown von Kossa stains in the resulting processed pictures were extracted with an Orange-G filter using CellProfiler (Version 4.2.8). An appropriate greyvalue threshold was applied and von Kossa stain positive area was quantified. Changes in ALP activity are reported as fold-of-control compared to differentiated and DMSO treated cells. *Statistical significance* was evaluated in GraphPad Prism 9 (GraphPad software) using ANOVA with Dunnett's multiple comparisons test. A p-value < 0.05 was considered statistically significant.

Computational procedures

General: Calculations were performed in Molecular Operating Environment (MOE, version 2024.06, Chemical Computing Group ULC, Montreal, QC, Canada) using default settings for each tool/function unless stated otherwise. Amber10:EHT was used as the default forcefield for all calculations.

Molecular Docking: Docking was performed using the X-ray structures 4g1d[1] and 2zxm[12] of the VDR LBD. Protonation states of the complexes were adjusted using the MOE QuickPrep tool. Compounds **4-10** were loaded separately in (*E*)- and (*Z*)-configurations in sdf format and prepared using the Energy minimize tool (with preserved unconstrained chirality) as well as the MOE Wash tool (protonation state dominant at pH 7.0, Rebuild 3D, preserved unconstrained chirality). Docking was performed using the following settings in the MOE Dock tool: receptor: receptor + solvent; site: ligand atoms; placement: Triangle Matcher; score: London dG; poses: 100; refinement: induced fit; refinement score: GBVI/WSA dG; poses: 10. Redocking of the crystallized ligand CD4720 (**3**) in VDR (pdb ID 4g1d[1]) resulted in a mean docking score of 13.04 (range 13.04 – 12.60, median 12.72) and a mean RMSD value of 0.701 (range 0.701 – 2.872, median 1.579). Redocking of the crystallized antagonist in VDR (pdb ID 2zxm[12]) resulted in a mean docking score of 12.77 (range 12.77 – 11.94, median 12.10) and a mean RMSD value of 0.264 (range 0.264 – 2.825, median 1.175). Note that the ligand bound in 2zxm does not match the annotated structure. The RMSD values between the docked poses and the crystallized ligand were calculated with the mol rmsd SVLscript in MOE.

*NMR Spectra of **4**-**10***

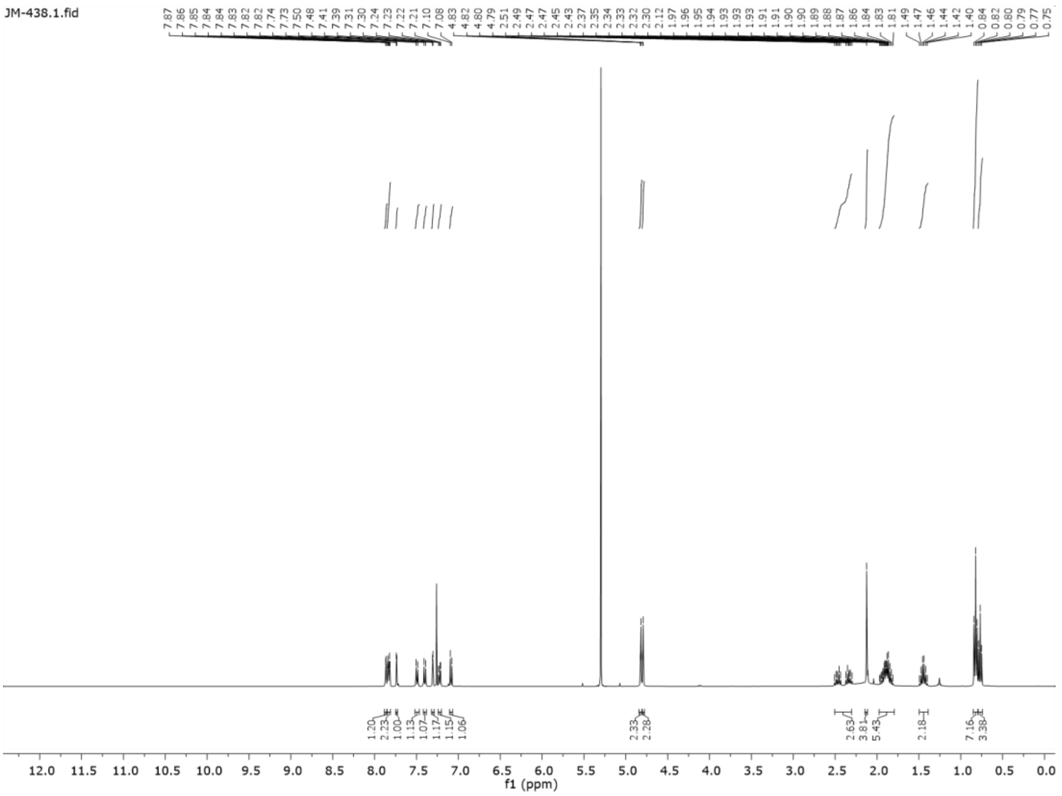

^{1}H NMR (400 MHz, $CDCl_3$) of compound **4**.

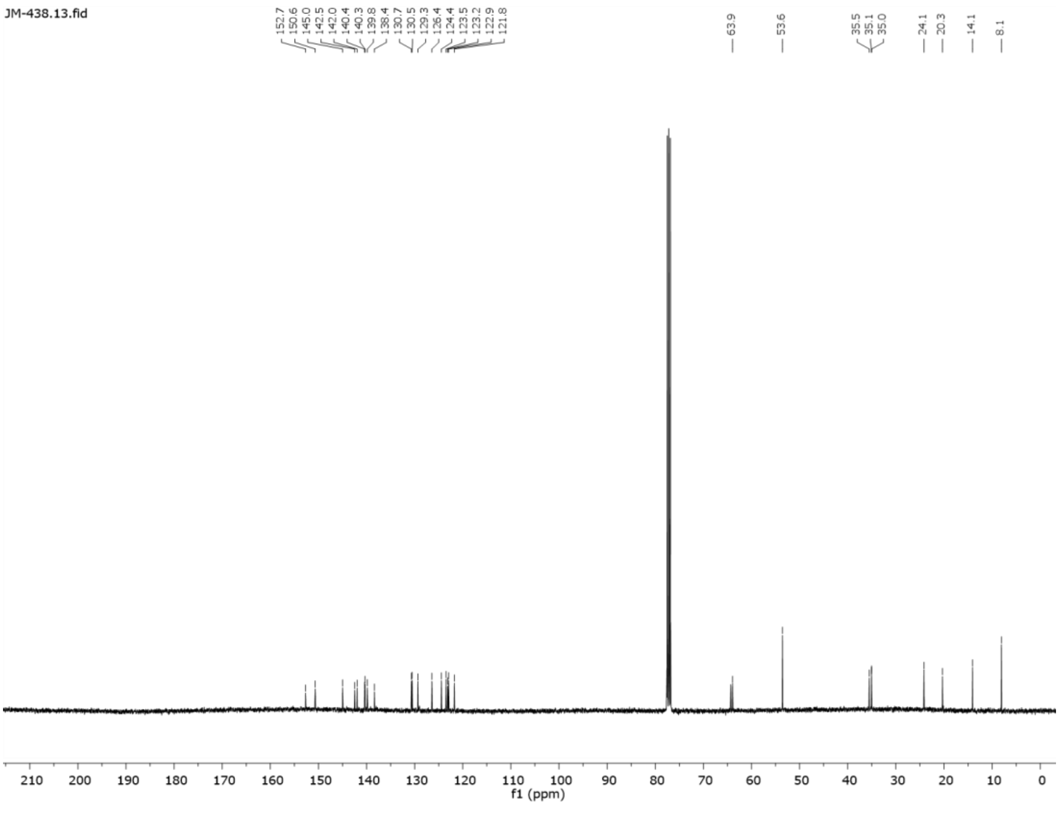

^{13}C NMR (101 MHz, $CDCl_3$) of compound **4**.

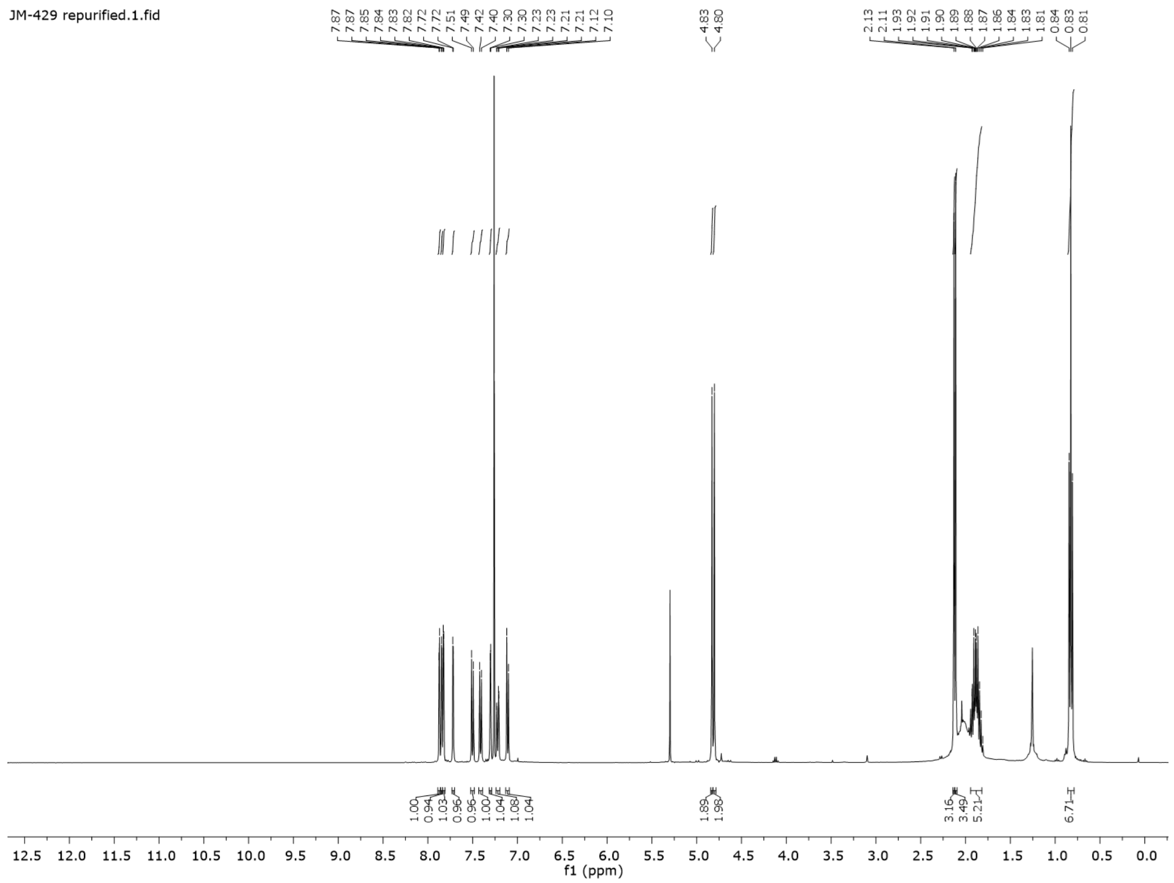

^{1}H NMR (400 MHz, $CDCl_3$) of compound **5**.

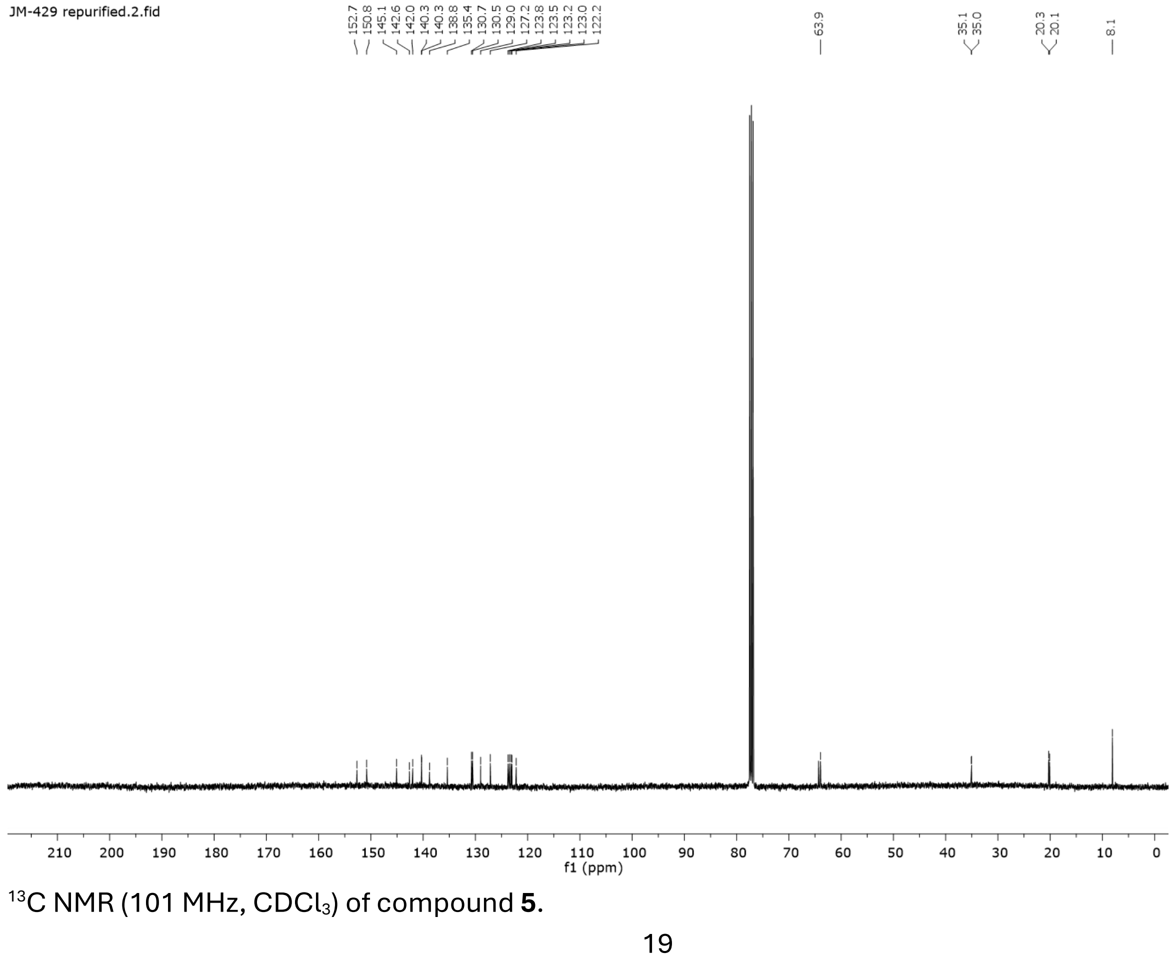

^{13}C NMR (101 MHz, $CDCl_3$) of compound **5**.

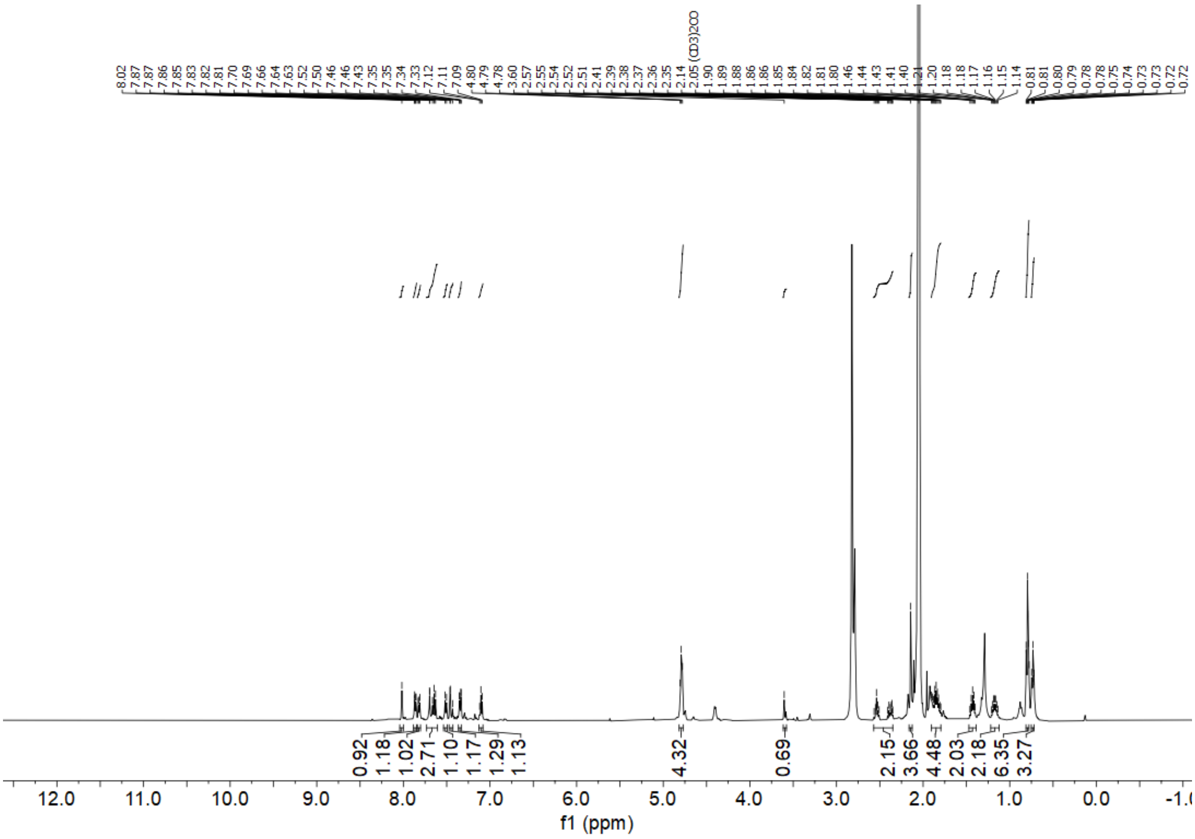

^{1}H NMR (500 MHz, acetone-d_6) of compound **6.**

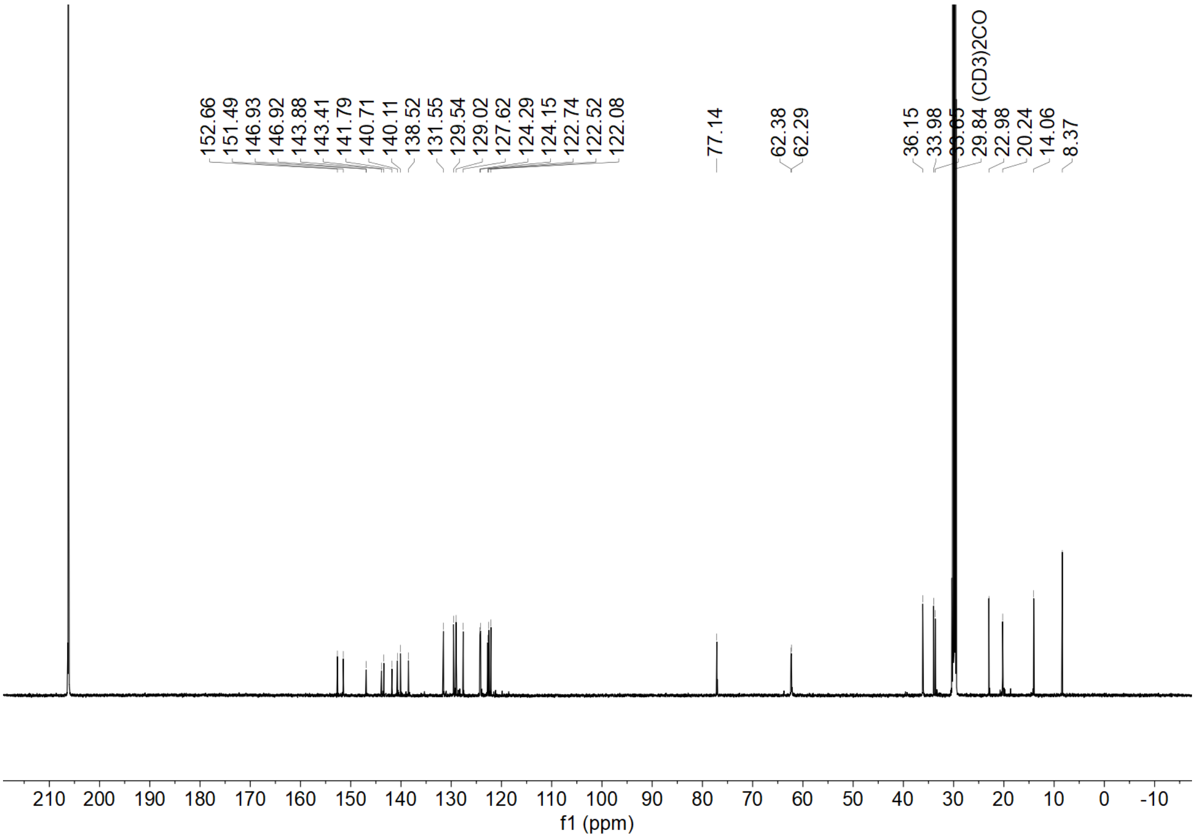

^{13}C NMR (151 MHz, acetone-d_6) of compound **6.**

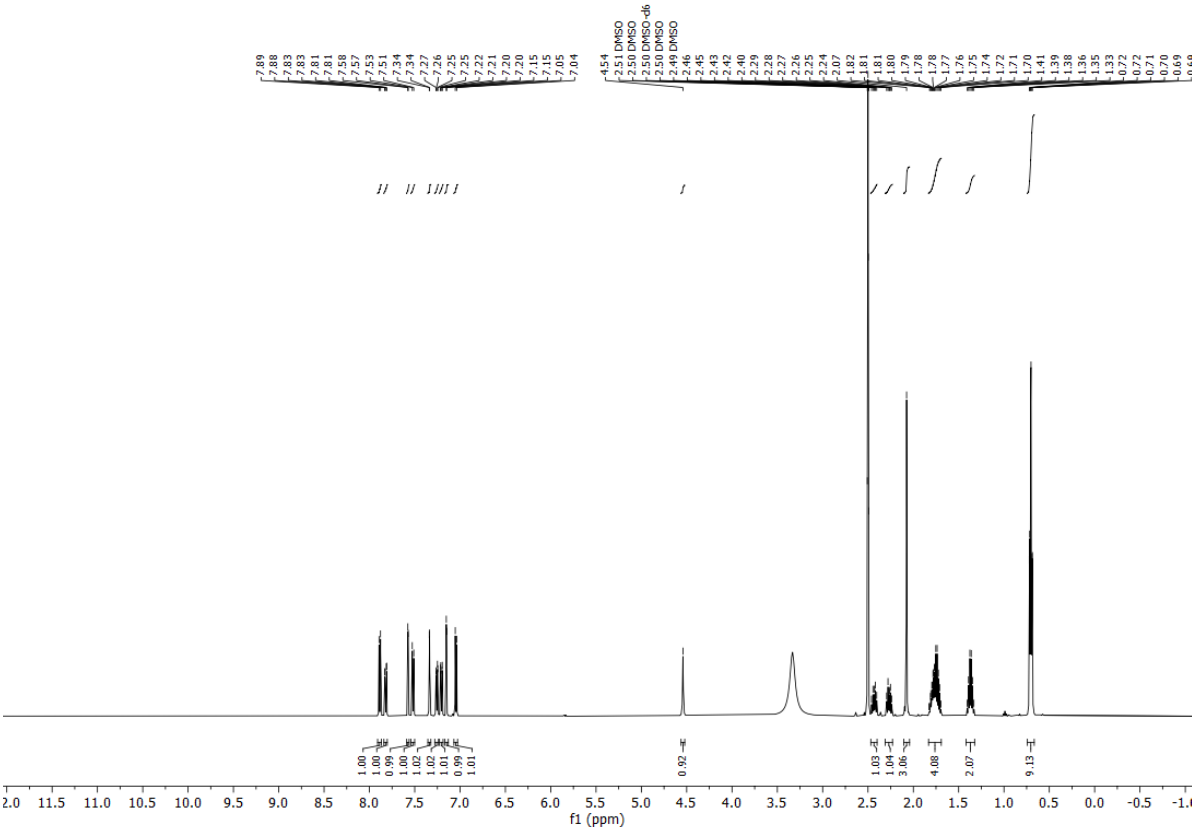

^{1}H NMR (500 MHz, DMSO-d_6) of compound **7**.

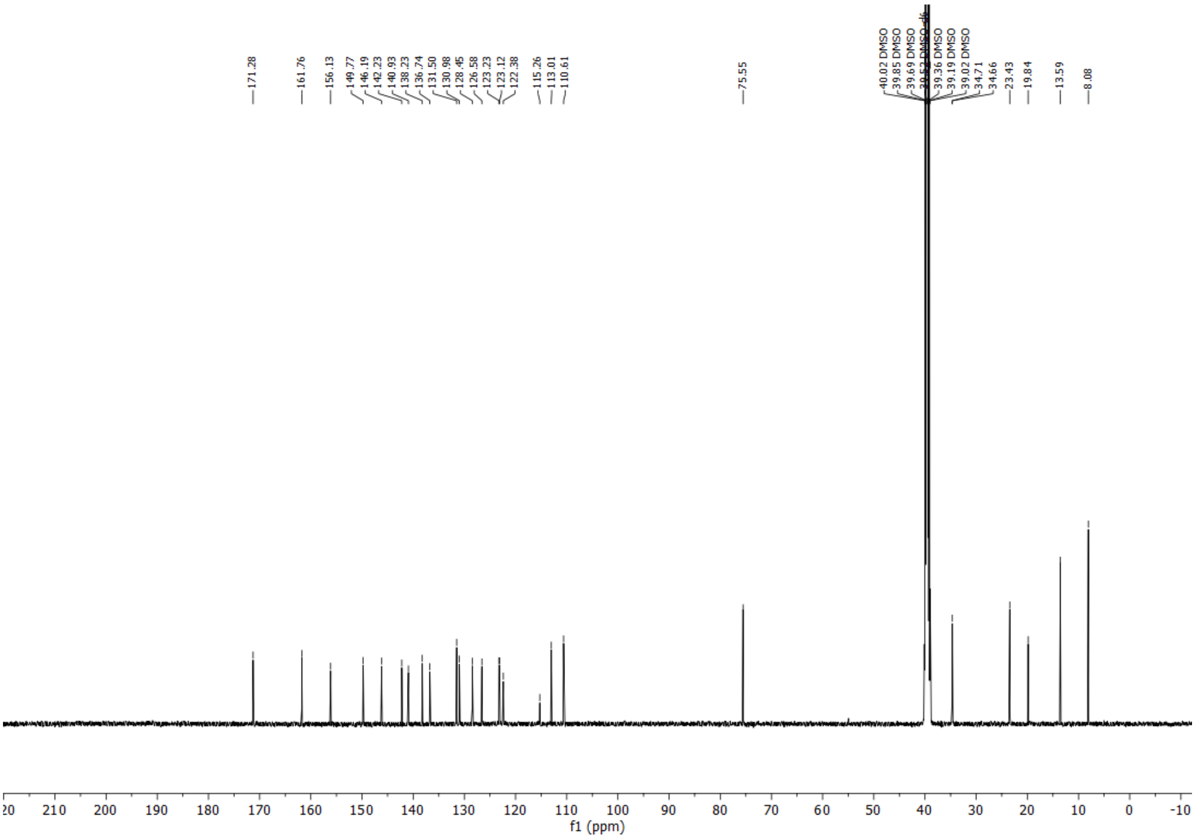

^{13}C NMR (126 MHz, DMSO-d_6) of compound **7**.

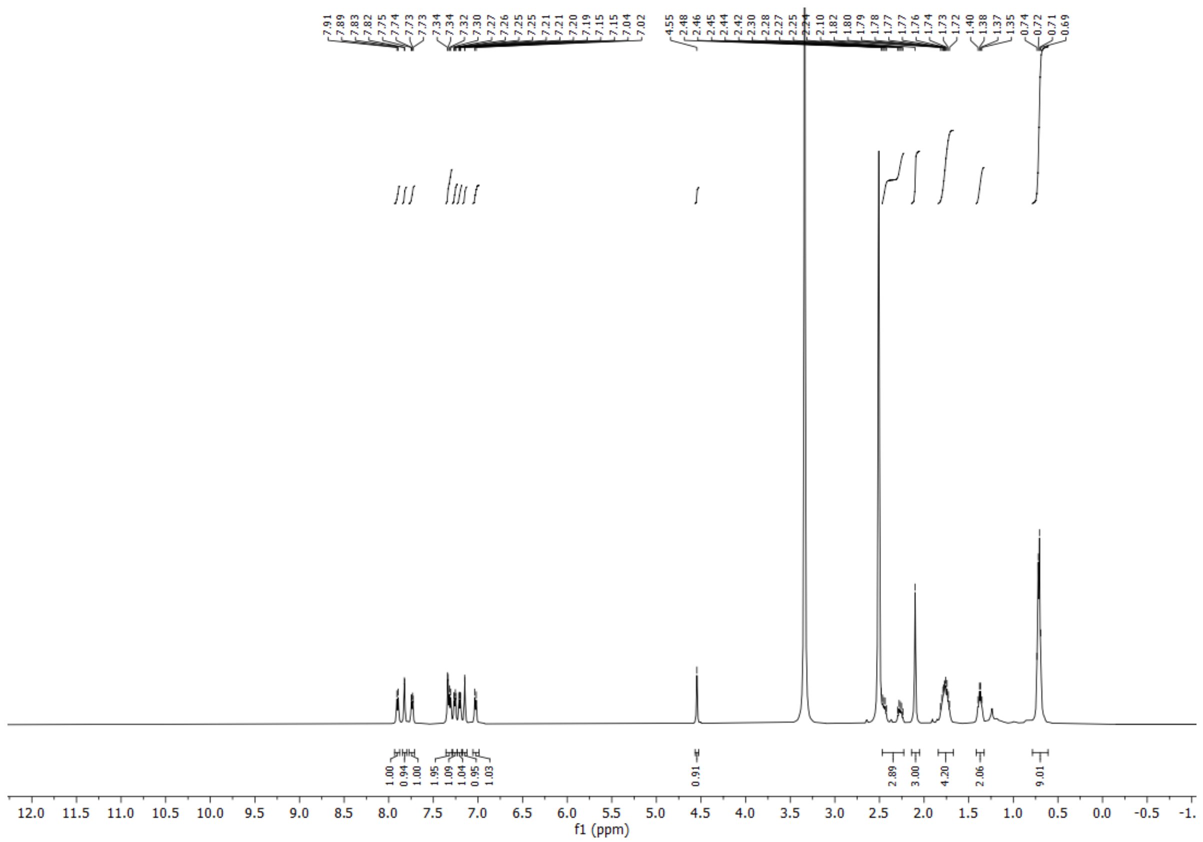

^{1}H NMR (500 MHz, DMSO) of compound **8**.

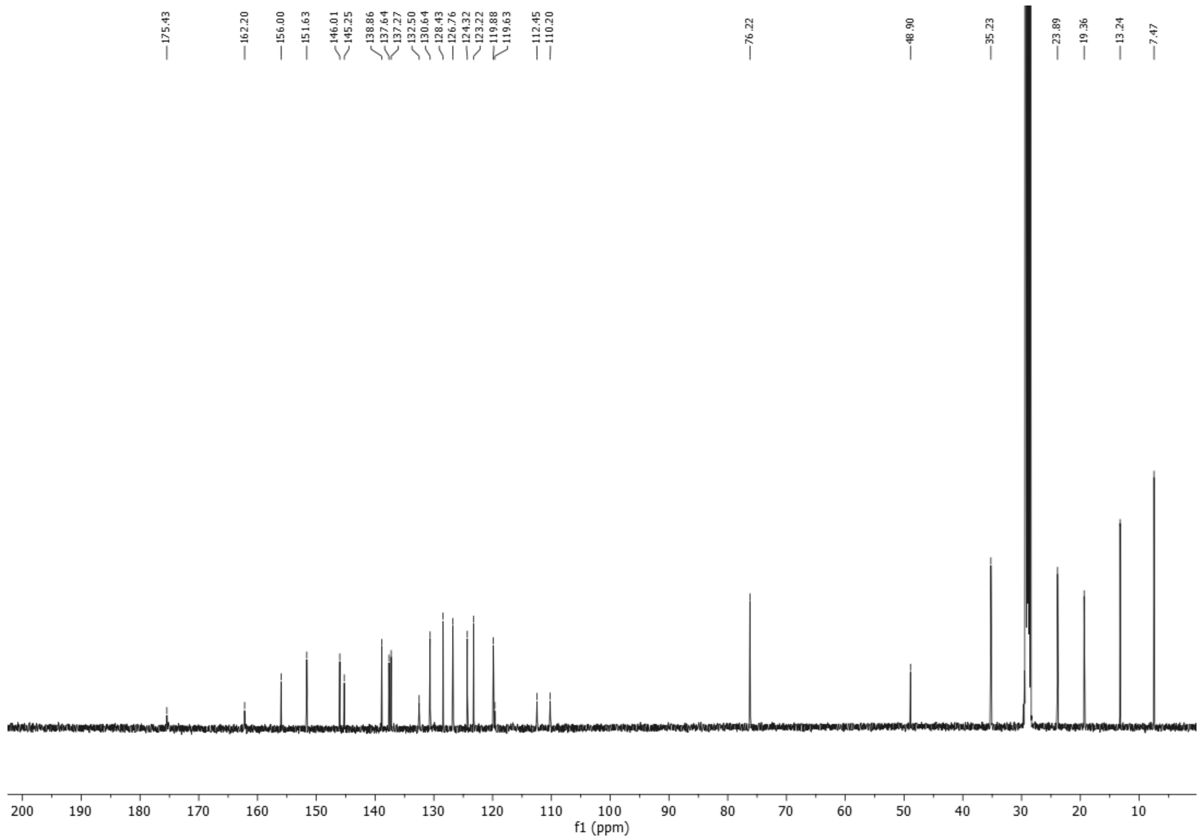

^{13}C NMR (126 MHz, acetone-d_6) of compound **8**.

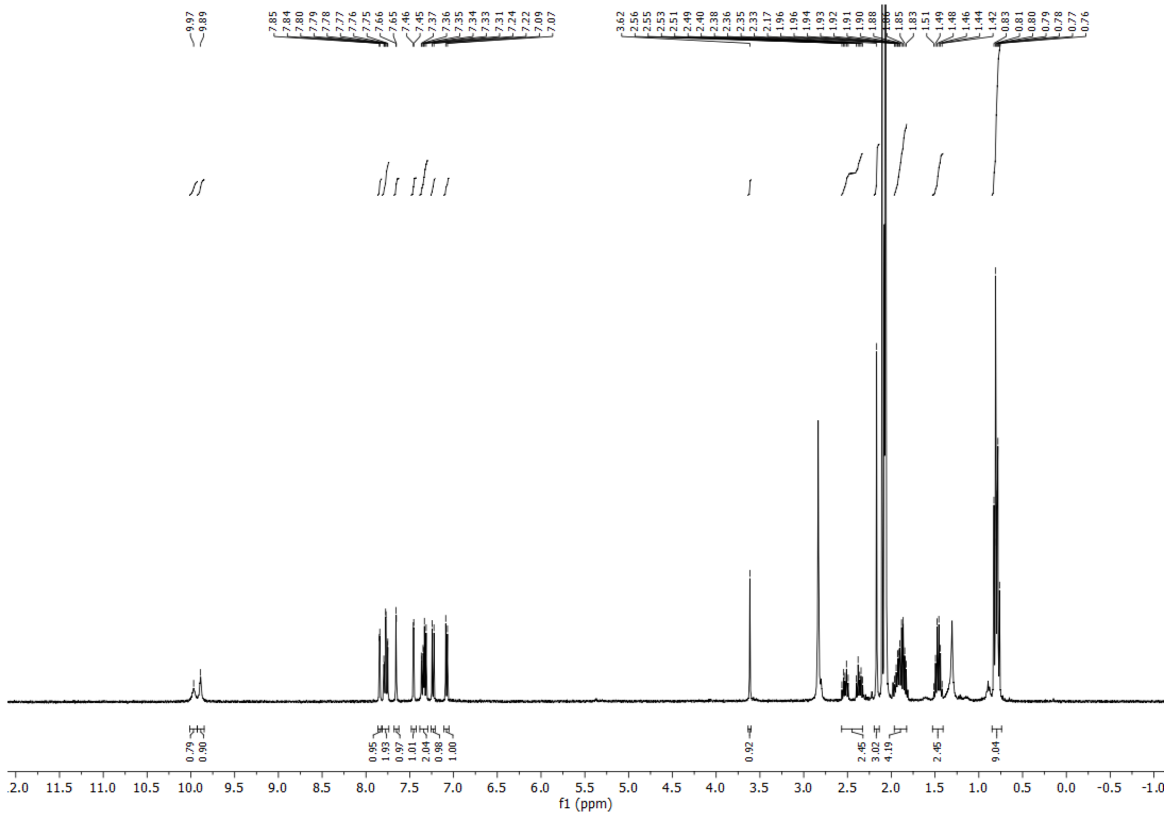

^{1}H NMR (400 MHz, acetone-d_6) of compound **9**.

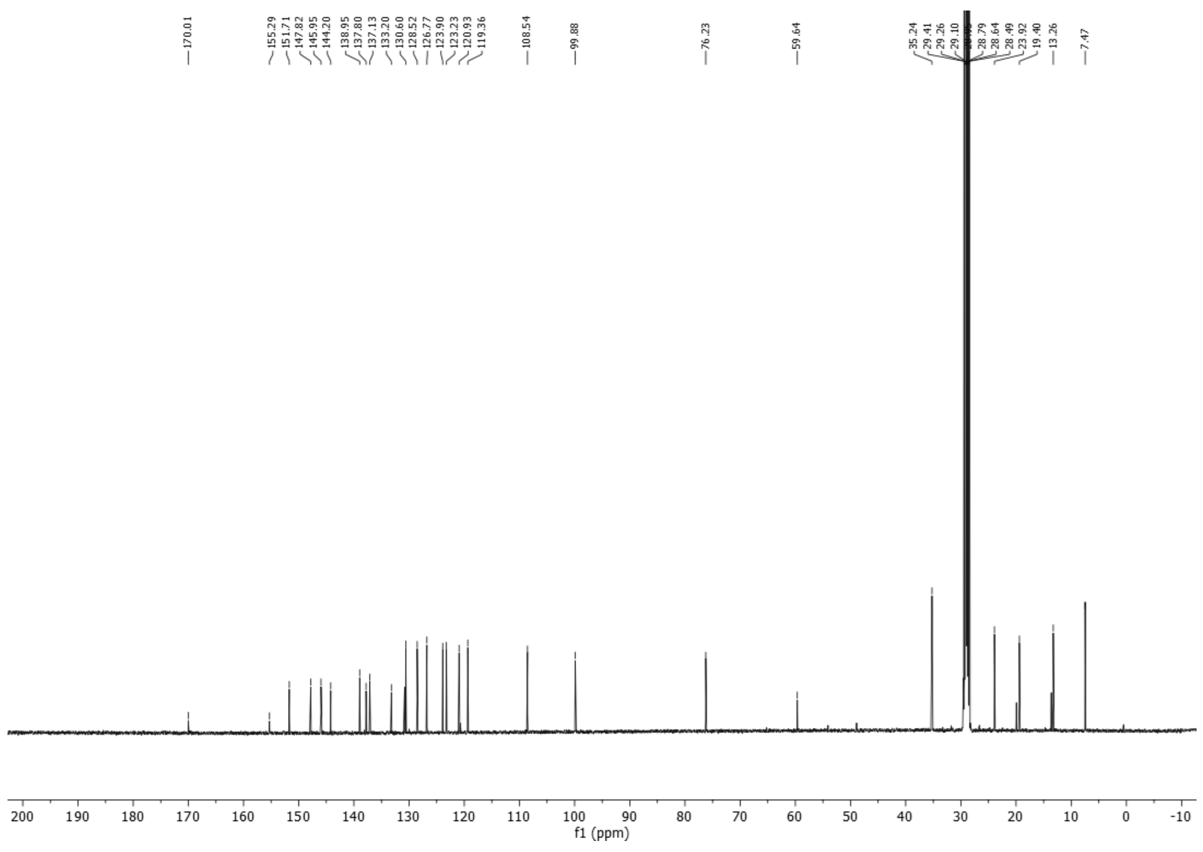

^{13}C NMR (101 MHz, acetone-d_6) of compound **9**.

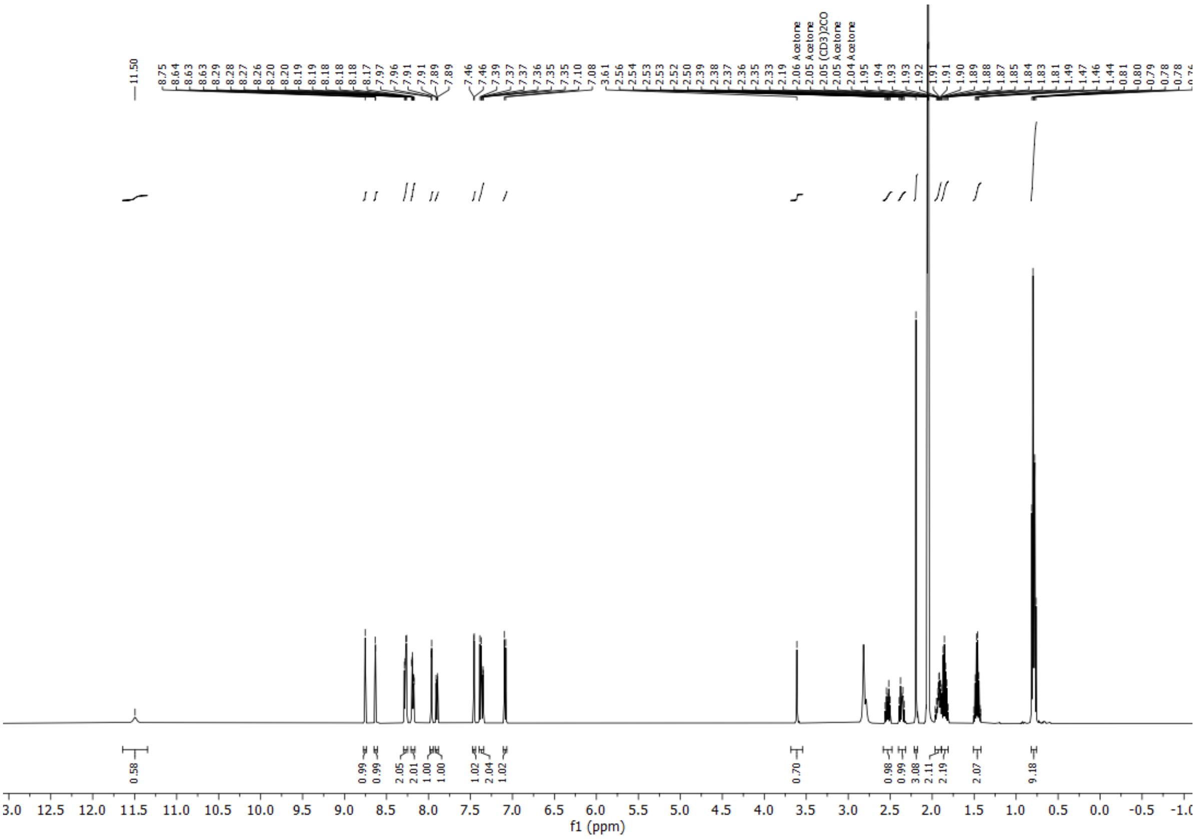

^{1}H NMR (500 MHz, acetone-d_6) of compound **10**.

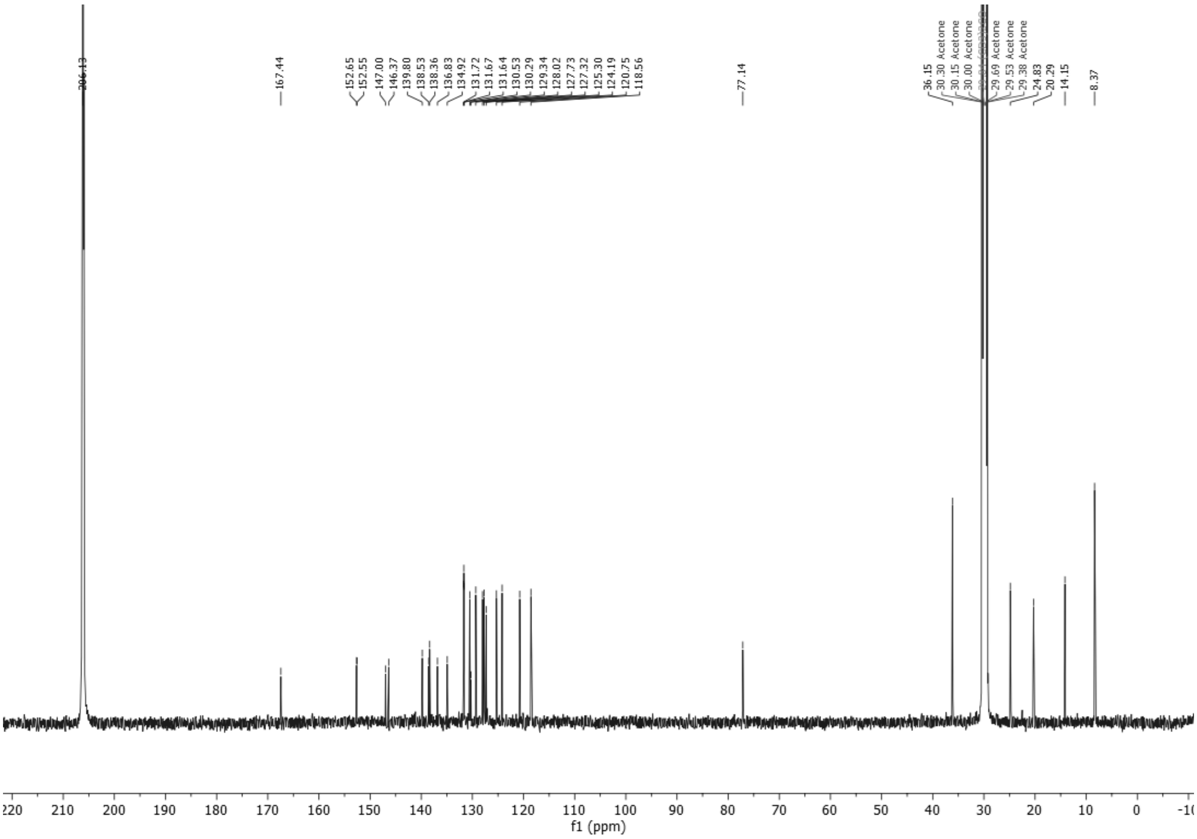

^{13}C NMR (126 MHz, acetone-d_6) of compound **10**.

Supplementary References

[1] F. Ciesielski, Y. Sato, Y. Chebaro, D. Moras, A. Dejaegere, N. Rochel, “Structural basis for the accommodation of bis- and tris-aromatic derivatives in vitamin D nuclear receptor” *J. Med. Chem.* **2012**, *55*, 8440–8449.

[2] G. F. Pauli, S. N. Chen, C. Simmler, D. C. Lankin, T. Gödecke, B. U. Jaki, J. B. Friesen, J. B. McAlpine, J. G. Napolitano, “Importance of purity evaluation and the potential of quantitative 1H NMR as a purity assay” *J. Med. Chem.* **2014**, *57*, 9220–9231.

[3] S. Jin, J. Wang, M. Li, B. Wang, “Synthesis, evaluation, and computational studies of naphthalimide-based long-wavelength fluorescent boronic Acid reporters” *Chemistry* **2008**, *14*, 2795–2804.

[4] E. Terranova, J. C. Pascal, *New 4-Hydroxyalkylphenyl Boronic Acid Derivatives, Useful in Synthesis of Non-Steroidal Vitamin D Analogs, Also Their New Intermediates*, **2003**, FR2864083A1.

[5] P. Demerseman, Pierre; Lechartier, Jean Pierre; Reynaud, Rene; Cheutin, Andree; Royer, Rene; Rumpf, “Research on the relations between the structures and the physicochemical properties of alkylphenols” *Bull. Soc. Chim. Fr.* **1963**, *11*, 2559–63.

[6] J. M. Bernardon, T. Biadatti, *ANALOGUES OF VITAMIN D*, **2001**, FR2833258A1.

[7] J. Heering, D. Merk, “Hybrid Reporter Gene Assays: Versatile In Vitro Tools to Characterize Nuclear Receptor Modulators” *Methods Mol. Biol.* **2019**, *1966*, 175–192.

[8] S. Arifi, J. A. Marschner, J. Pollinger, L. Isigkeit, P. Heitel, A. Kaiser, L. Obeser, G. Höfner, E. Proschak, S. Knapp, A. Chaikuad, J. Heering, D. Merk, “Targeting the Alternative Vitamin E Metabolite Binding Site Enables Noncanonical PPARγ Modulation” *J. Am. Chem. Soc.* **2023**, *145*, 14802–14810.

[9] M. Borowiak, W. Nahaboo, M. Reynders, K. Nekolla, P. Jalinot, J. Hasserodt, M. Rehberg, M. Delattre, S. Zahler, A. Vollmar, D. Trauner, O. Thorn-Seshold, “Photoswitchable Inhibitors of Microtubule Dynamics Optically Control Mitosis and Cell Death” *Cell* **2015**, *162*, 403–411.

[10] W. Zhang, A. W. Lohman, Y. Zhuravlova, X. Lu, M. D. Wiens, H. Hoi, S. Yaganoglu, M. A. Mohr, E. N. Kitova, J. S. Klassen, P. Pantazis, R. J. Thompson, R. E. Campbell, “Optogenetic control with a photocleavable protein, Phocl” *Nat. Methods* **2017**, *14*, 391–394.

[11] S. Wolbank, G. Stadler, A. Peterbauer, A. Gillich, M. Karbiener, B. Streubel, M. Wieser, H. Katinger, M. Van Griensven, H. Redl, C. Gabriel, J. Grillari, R. Grillari-Voglauer, “Telomerase immortalized human amnion- and adipose-derived mesenchymal stem cells: maintenance of differentiation and immunomodulatory characteristics” *Tissue Eng. Part A* **2009**, *15*, 1843–1854.

[12] Y. Inaba, N. Yoshimoto, Y. Sakamaki, M. Nakabayashi, T. Ikura, H. Tamamura, N. Ito, M. Shimizu, K. Yamamoto, “A new class of vitamin D analogues that induce structural rearrangement of the ligand-binding pocket of the receptor” *J. Med. Chem.* **2009**, *52*, 1438–1449.
